# Installation of O-glycan sulfation capacities in human HEK293 cells for display of sulfated mucins

**DOI:** 10.1016/j.jbc.2021.101382

**Published:** 2021-12-24

**Authors:** Lingbo Sun, Andriana Konstantinidi, Zilu Ye, Rebecca Nason, Yuecheng Zhang, Christian Büll, Barbro Kahl-Knutson, Lars Hansen, Hakon Leffler, Sergey Y. Vakhrushev, Zhang Yang, Henrik Clausen, Yoshiki Narimatsu

**Affiliations:** 1 Departments of Cellular and Molecular Medicine, Faculty of Health Sciences, Copenhagen Center for Glycomics, University of Copenhagen, Copenhagen N, Denmark; 2 Medical College of Yan’an University, Yan’an University, Yan’an, Shaanxi Province, China; 3 Department of Biomedical Sciences, Faculty of Health and Society, Malmö University, Malmö, Sweden; 4 Department of Laboratory Medicine, Section MIG, Lund University BMC-C1228b, Lund, Sweden

**Keywords:** glycosylation, O-glycans, sulfation, sulfated-T, sulfated-Tn, mucins1, BSA, bovine serum albumin, FPKM, fragments per kilobase of transcript per million mapped reads, GAG, glycosaminoglycan, GAL-4, galectin-4, gRNA, guide RNA, HCD, higher-energy collisional dissociation, HEK293, human embryonic kidney 293 cells, KI, knockin, KO, knockout, KS, keratan sulfate, mAb, monoclonal antibody, MS, mass spectrometry, PNA, peanut agglutinin, RT, room temperature, siglec, sialic acid–binding immunoglobulin-like lectin, TR, tandem repeat

## Abstract

The human genome contains at least 35 genes that encode Golgi sulfotransferases that function in the secretory pathway, where they are involved in decorating glycosaminoglycans, glycolipids, and glycoproteins with sulfate groups. Although a number of important interactions by proteins such as selectins, galectins, and sialic acid–binding immunoglobulin-like lectins are thought to mainly rely on sulfated O-glycans, our insight into the sulfotransferases that modify these glycoproteins, and in particular GalNAc-type O-glycoproteins, is limited. Moreover, sulfated mucins appear to accumulate in respiratory diseases, arthritis, and cancer. To explore further the genetic and biosynthetic regulation of sulfated O-glycans, here we expanded a cell-based glycan array in the human embryonic kidney 293 (HEK293) cell line with sulfation capacities. We stably engineered O-glycan sulfation capacities in HEK293 cells by site-directed knockin of sulfotransferase genes in combination with knockout of genes to eliminate endogenous O-glycan branching (core2 synthase gene *GCNT1*) and/or sialylation capacities in order to provide simplified substrates (core1 Galβ1–3GalNAcα1–O-Ser/Thr) for the introduced sulfotransferases. Expression of the galactose 3-*O*-sulfotransferase 2 in HEK293 cells resulted in sulfation of core1 and core2 O-glycans, whereas expression of galactose 3-*O*-sulfotransferase 4 resulted in sulfation of core1 only. We used the engineered cell library to dissect the binding specificity of galectin-4 and confirmed binding to the 3-*O*-sulfo-core1 O-glycan. This is a first step toward expanding the emerging cell-based glycan arrays with the important sulfation modification for display and production of glycoconjugates with sulfated O-glycans.

Sulfated mucoproteins were originally characterized by use of the periodic acid-Schiff and toluidine blue staining method (1). Sulfated mucins were isolated from gastrointestinal, bronchial, reproductive, and other tissues (2, 3, 4), and sulfation was analyzed by ^35^S-radiolabeling and chromatography (5, 6). With the development of mass spectrometry (MS), insight into the detailed structures of sulfated glycans on N-glycoproteins and O-glycoproteins has evolved. Khoo *et al.* (7, 8) established a highly selective enrichment and sensitive MS mapping method for sulfated N-glycans and/or O-glycans based on MALDI–MS/MS analysis of permethylated glycans in negative ion mode. The MALDI–MS/MS was further extended to more comprehensive and targeted LC–MS/MS analysis, resulting in rapid selection and sequencing of sulfated glycans at high resolution (9, 10, 11, 12, 13). Recently, a higher-energy collisional dissociation (HCD) fragmentation technique was used to identify sulfated O-glycans on the mucins5B providing confident identification of larger sulfated glycans (14). A number of sulfated O-glycans were identified in the mouse secondary lymph nodes by MALDI–MS mapping of fractionated permethylated O-glycans (7). Multiple reaction monitoring approach was applied to decipher O-glycan isomers from lubricin using porous graphitized carbon chromatography in the negative ion mode, and a large number of isomeric O-glycans with sulfate and sialic acid groups were identified on lubricin (15) and the salivary mucin7 (MG2) (16).

Mucin-type (GalNAc-type) O-glycosylation (hereafter, O-glycosylation) is one of the most common and diverse types of protein glycosylation, and more than 85% of proteins trafficking the secretory pathway are predicted to acquire O-glycans (17). The most common O-glycan structures are sialylated core1 structures (NeuAcα2–3Galβ1–3[NeuAcα2–6]_+/−_GalNAcα1–O-Ser/Thr, also known as monosialyl-T and disialyl-T, mST, and dST) and branched and sialylated core2 structures (NeuAcα2–3Galβ1–3[NeuAcα2–3Galβ1–3/4GlcNAcβ1–6]GalNAcα1–O-Ser/Thr), whereas core3 and core4 structures are selectively found in epithelial cells of the gastrointestinal tract (18). More complex structures with elongated poly-LacNAc, fucose, and other blood group–related epitopes are produced in different cell types, and sulfation of Gal, GlcNAc, and GalNAc residues has been reported on O-glycans (2, 11, 15).

Knowledge of the nature and biological functions of sulfated mucins is still limited, and the genetic and biosynthetic regulation of sulfation of mucin-type O-glycans is poorly understood (19). Sulfated O-glycans are ligands for members of the selectin, sialic acid–binding immunoglobulin-like lectins (Siglecs), and galectin families (20, 21, 22). For example, L-selectin specifically recognizes 6-*O*-sulfated SLe^x^ (NeuAcα2–6Galβ1–4[Fucα1–3;6-sulfo]GlcNAc) (23), CD22/Siglec-2 preferentially binds 6-*O*-sulfated LacNAc (NeuAcα2–6Galβ1–4[6-sulfo]GlcNAc) on N-glycans and O-glycans on B cells (24), and the 6-*O*-sulfated and sialylated LacNAc was recently discovered as the binding epitope for CD33/Siglec-3, which is associated with Alzheimer's disease (25). A number of monoclonal antibodies (mAbs) directed against sulfated glycan epitopes have been generated, for example, the MECA-79 mAb that defines endothelial cells lining postcapillary high endothelial venules recognizes a 6-*O*-sulfated extended core1 O-glycan (Galβ1–4[6-sulfo]GlcNAcβ1–3Galβ1–3GalNAcα1–O-Ser/Thr) (26, 27), the human natural killer-1 mAb that recognizes a 3-*O*-sulfated glycan epitope (3-sulfo-GlcA1β1–3Galβ1–4GlcNAc) found on glycolipids, N-glycans, and O-glycans (28, 29); and the M-DC8 mAb that recognizes 6-sulfo LacNAc structure (Galβ1–4[6-sulfo]GlcNAc) associated with proinflammatory dendritic cells (30).

The sulfotransferases involved in sulfation of O-glycans are poorly defined. A total of 50 sulfotransferase genes are found in the human genome, and these include 13 cytosolic sulfotransferases that function with proteins, lipids, and steroids in detoxification (31, 32), two protein tyrosine sulfotransferases (33, 34), and 35 Golgi sulfotransferases that use glycans and glycosaminoglycans (GAGs) as acceptor substrates (35). Studies of the latter enzymes have mainly been performed with *in vitro* enzyme assays, and their functions in sulfation of O-glycans remain largely predictions (please see Table S1 with references).

Here, we took a first step toward stable genetic installation of sulfation capacities for O-glycans in the human embryonic kidney 293 cell line (HEK293) to display and produce sulfated variants of the cancer-associated T (Galβ1–3GalNAcα1–O-Ser/Thr) and Tn (GalNAcα1–O-Ser/Thr) O-glycans on human mucins (36, 37). Genetic engineering of the glycosylation capacities in a cell line can be used to dissect biosynthesis and genetic regulation of specific glycosylation features, and with more comprehensive and systematic engineering to develop a cell-based glycan array that displays the glycome on cells for biological interrogation (38, 39). We obtained engineered HEK293 cells that display and produce 3-*O*-sulfated core1 and core2 O-glycans by installation of the GAL3ST2 and GAL3ST4 sulfotransferases and used these to express small GPF-tagged reporters containing tandem repeat (TR) sequences derived from human mucins (40). We used the platform to dissect the binding specificity of galectin-4 (GAL-4) for 3-*O*-sulfo-core1 O-glycans.

## Results

### Prediction of human sulfotransferases serving simple O-glycans

**Figure 1:**
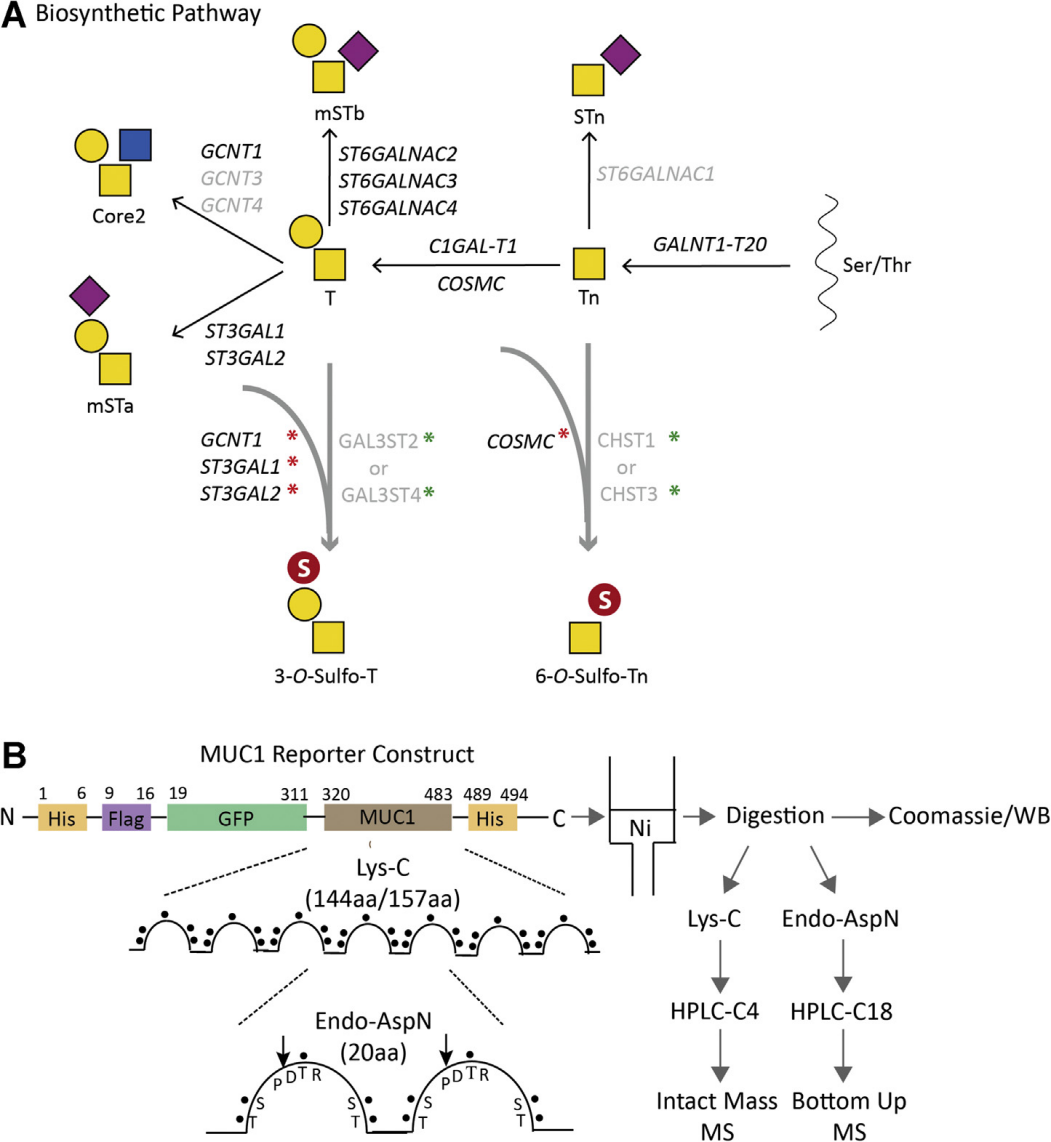
**Graphic depiction of engineering strategy and experimental flow for installation of O-glycan sulfation in HEK293 cells.***A*, biosynthesis and genetic regulation of GalNAc-type O-glycosylation highlighting genes are expressed in HEK293 cells (*black text*), and the engineering strategy performed with KO (*capitalized and italicized with red asterisk*) and individual KI of sulfotransferase complementary DNA (*capitalized and nonitalicized with green asterisk*). *B*, illustration of the secreted mucin1 TR reporter containing 6.5 × 20-mer TRs derived from the TR domain of human mucin1, and the Lys-C and endo-AspN digestion strategy and purification workflow for MS analysis. Structures of glycans are shown with symbols drawn according to the Symbol Nomenclature for Glycans format (74). HEK293, human embryonic kidney 293 cell line; KI, knockin; TR, tandem repeat.

Fig. S1 provides an overview of identified human Golgi sulfotransferase genes and their predicted roles in sulfation of glycolipids, glycoproteins, and GAGs (19, 38, 41). The largest groups of sulfotransferase paralogs are predicted to act in the biosynthesis of GAGs, whereas three groups (CHST8–10, CHST1–7, and GAL3ST1–4) are predicted to serve glycolipids and glycoproteins, including keratan sulfate (KS). These latter groups of sulfotransferases cannot be unambiguously assigned to specific types of glycoconjugates and glycosylation pathways, except for GAL3ST1 that only serves glycolipids (42). Reviewing the available reported data on substrate specificities of the remaining 13 sulfotransferases (please see references in Table S1), we predicted that GAL3ST2 and GAL3ST4 were the primary candidates for directing 3-*O*-sulfation of the Gal residue in the simple core1 O-glycan (T) structure, and that CHST1 and CHST3 potentially directed 6-*O*-sulfation of core1 and/or the GalNAc residue of the Tn *O*-glycan (Fig. 1*A*). GAL3ST2 was previously in *in vitro* assays found to exhibit 3-*O*-sulfotransferase activity with both Galβ1–3GalNAc-*O*-benzyl and Galβ1–3/4GlcNAc disaccharide substrates, whereas GAL3ST4 was shown to only use the Galβ1–3GalNAc-*O*-benzyl substrate (43, 44). The CHST1 6-*O*-sulfotransferase functions not only in KS biosynthesis (45) but also in 6-*O*-sulfation of Gal residues in extended core1 or with GalNAc in sialylated core1 (mSTa) as demonstrated in *in vitro* studies in Chinese hamster ovary cells (36). CHST3 appears to be the evolutionary closest sulfotransferase to CHST1 and reported to function in biosynthesis of chondroitin sulfate and KS (46). To install sulfation of ST, T, and Tn O-glycans, we therefore considered these four sulfotransferases (Fig. 1*A*).

### Engineering strategy for 3-*O*-sulfated core1 O-glycans

The HEK293 cell line is widely used for recombinant expression of glycoproteins, and the wildtype cell has capacity for production of core1 and core2 O-glycans capped by sialic acids (Fig. S2). RNA-Seq transcriptomics analysis of suspension HEK293 wildtype (HEK293^WT^) cells indicated that most of the sulfotransferases predicted to serve glycoproteins, including *GAL3ST2* and *GAL3ST4*, were not expressed (fragments per kilobase of transcript per million mapped reads [FPKMs] <1) (Fig. S3). We used a targeted knockin (KI) strategy to introduce GAL3ST2 or GAL3ST4 in combination with knockout (KO) of competing glycosyltransferases using a validated guide RNA (gRNA) targeting library previously described (41) (Figs. 1*A* and S2). We first installed GAL3ST2 or GAL3ST4 to generate HEK293^KI GAL3ST2^ and HEK293^KI GAL3ST4^ and then eliminated core2 O-glycan branching by KO of *GCNT1* to establish HEK293^KO^ ^*GCNT1*, KI^ ^GAL3ST2^ and HEK293^KO^ ^*GCNT1*, KI GAL3ST4^. Subsequently, we eliminated sialylation by double KO of *ST3GAL1*/*2* encoding the main α2–3 sialyltransferases capping core1 O-glycans to generate HEK293^KO^ ^*GCNT1*,^ ^*ST3GAL1/2*, KI GAL3ST2^ and HEK293^KO^ ^*GCNT1*,^ ^*ST3GAL1/2*, KI GAL3ST4^ cell lines. This engineering strategy is predicted to provide homogenous unsubstituted core1 substrate for the introduced sulfotransferases and optimal conditions for sulfation. Note that we did not eliminate α2–6 sialyltransferases (*ST6GALNAC1–6*) that functions on core1 to form dST and monosialyl-T (mSTb) with or without the terminal α2–3 linked sialic acid. We also used the same KO designs without installation of sulfotransferase as counterpart to generate HEK293^KO^ ^*GCNT1*^ and HEK293^KO^ ^*GCNT1, ST3GAL1/2*^ (Table S2).

### Engineering strategy for 6-*O*-sulfated Tn O-glycans

6-*O*-sulfated core1 O-glycans have been reported on the mucin1 and mucin7 with involvement of CHST1 (36, 37), whereas a putative aberrant 6-*O*-sulfated Tn O-glycan has not been reported to our knowledge. The RNA-Seq transcriptomics analysis suggested that *CHST1* (FPKM < 1) and *CHST3* (FPKM <5) were not or only weakly expressed in HEK293^WT^ (Fig. S3), so we installed CHST1 or CHST3 to generate HEK293^KI CHST1^ and HEK293^KI CHST3^. We previously reported that KO of *COSMC* in HEK293 cells results in homogeneous Tn glycosylation without detectable STn (38) (Fig. 1*A*), and we therefore eliminated COSMC to provide homogenous Tn substrates (HEK293^KO^ ^*COSMC*, KI CHST1^, HEK293^KO^ ^*COSMC*, KI CHST3^) for sulfo-Tn (Table S2).

### Evaluation of sulfation capacities in engineered cell lines

**Figure 2:**
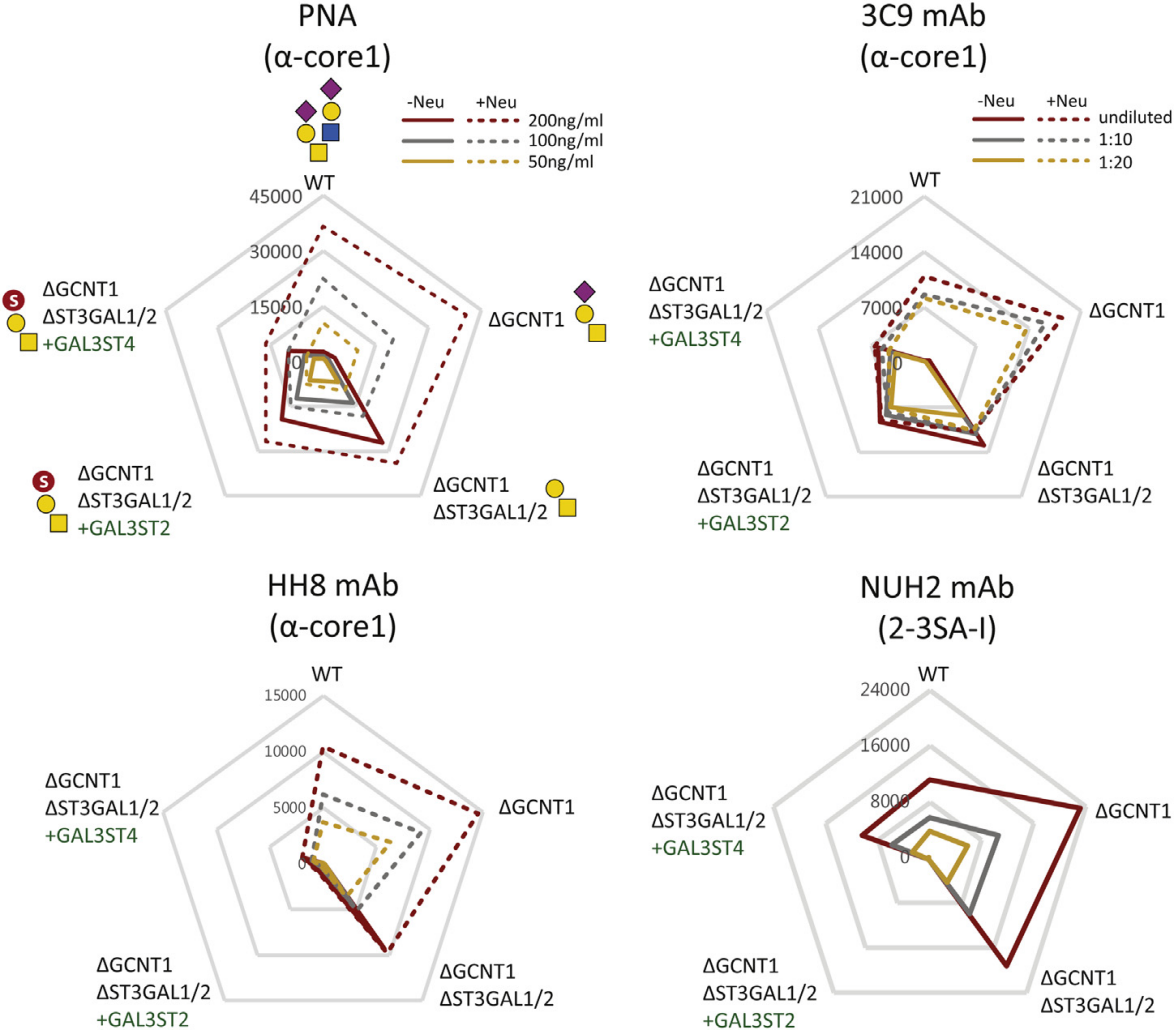
**Flow cytometry analysis of glycoengineered HEK293 cells with lectins and mAbs.** HEK293 cells stably engineered with KO (Δ) and KI (+) as indicated were analyzed with PNA lectin and mAbs 3C9, HH8, and NUH2 at different concentrations (*color coded*). *Radar charts* show mean fluorescence intensities (MFIs), and *solid*/*dashed lines* represent binding with and without pretreatment with neuraminidase. *Charts* represent single experiments, and independent experiments were performed at least three times with similar results. The predicted glycan structures produced by the glycoengineered HEK293 cells are shown in *first panel* (PNA). HEK293, human embryonic kidney 293 cell line; KI, knockin; mAb, monoclonal antibody; PNA, peanut agglutinin.

To evaluate effects of the engineering, we first explored lectin and antibody profiling of live nonpermeabilized cells. Exposure of core1 T O-glycans with lectin (peanut agglutinin [PNA]) and mAbs (3C9, HH8) (47) was used to evaluate the effects of KI of GAL3ST2 and GAL3ST4 (Fig. 2). HEK293^WT^ and HEK293^KO^ ^*GCNT1*^ cells did not expose T O-glycans, but strong binding of all reagents was induced by neuraminidase treatment. Combining KO of *GCNT1* and *ST3GAL1/2* (HEK293^KO^ ^*GCNT1*,^ ^*ST3GAL1/2*^) also induced the same strong binding with all anti-T reagents, and the binding was not affected by neuraminidase treatment for the mAbs but slightly for PNA. In contrast, HEK293^KO^ ^*GCNT1*,^ ^*ST3GAL1/2*, KI GAL3ST2^ or to greater extent HEK293^KO^ ^*GCNT1*,^ ^*ST3GAL1/2*, KI GAL3ST4^ with KI of sulfotransferases showed reduced or no binding. These reaction patterns were not substantially affected by neuraminidase pretreatment suggesting that the GAL3ST4 and to a lesser extent GAL3ST2 were effective in capping exposed core1 T O-glycans with sulfate.

Previously, GAL3ST2 was suggested to preferentially function with Galβ1–4GlcNAc (LacNAc) substrates compared with core1 (43). We therefore took advantage of the mAb NUH2 directed against the disialyl-I branched glycolipid with α2–3Neu5Ac capping and reactivity with HEK293^WT^ cells (38, 48). KO of *GCNT1* slightly enhanced binding of NUH2 (approximately twofold) for unknown reasons, but KI of GAL3ST2 completely abrogated binding of NUH2 similar level as the KO of I-branching *GCNT2* gene (38), whereas KI of GAL3ST4 reduced the binding to levels comparable to HEK293^WT^ cells (Fig. 2). This suggests that GAL3ST2 efficiently sulfates the I branched glycolipid and presumably competes with sialylation by ST3GAL6 (38).

To evaluate if CHST1 and CHST3 sulfated Tn O-glycans, we monitored exposure of Tn with the vicia villosa agglutinin lectin and the mAb 5F4. HEK293^WT^ cells were barely reactive with these, whereas HEK293^KO^ ^*COSMC*^ cells were strongly reactive, and this was abolished by KI of ST6GALNAC1 that results in STn (NeuAcα2–6GalNAcα1–O-Ser/Thr) O-glycans as expected (Fig. S4*A*). KI of CHST1 and CHST3 did not produce substantial changes, suggesting that sulfation of Tn by these sulfotransferases even in the absence of competing galactosylation and sialylation does not occur or is limited.

### Sulfation of recombinant expressed secreted mucin TR reporters

**Figure 3:**
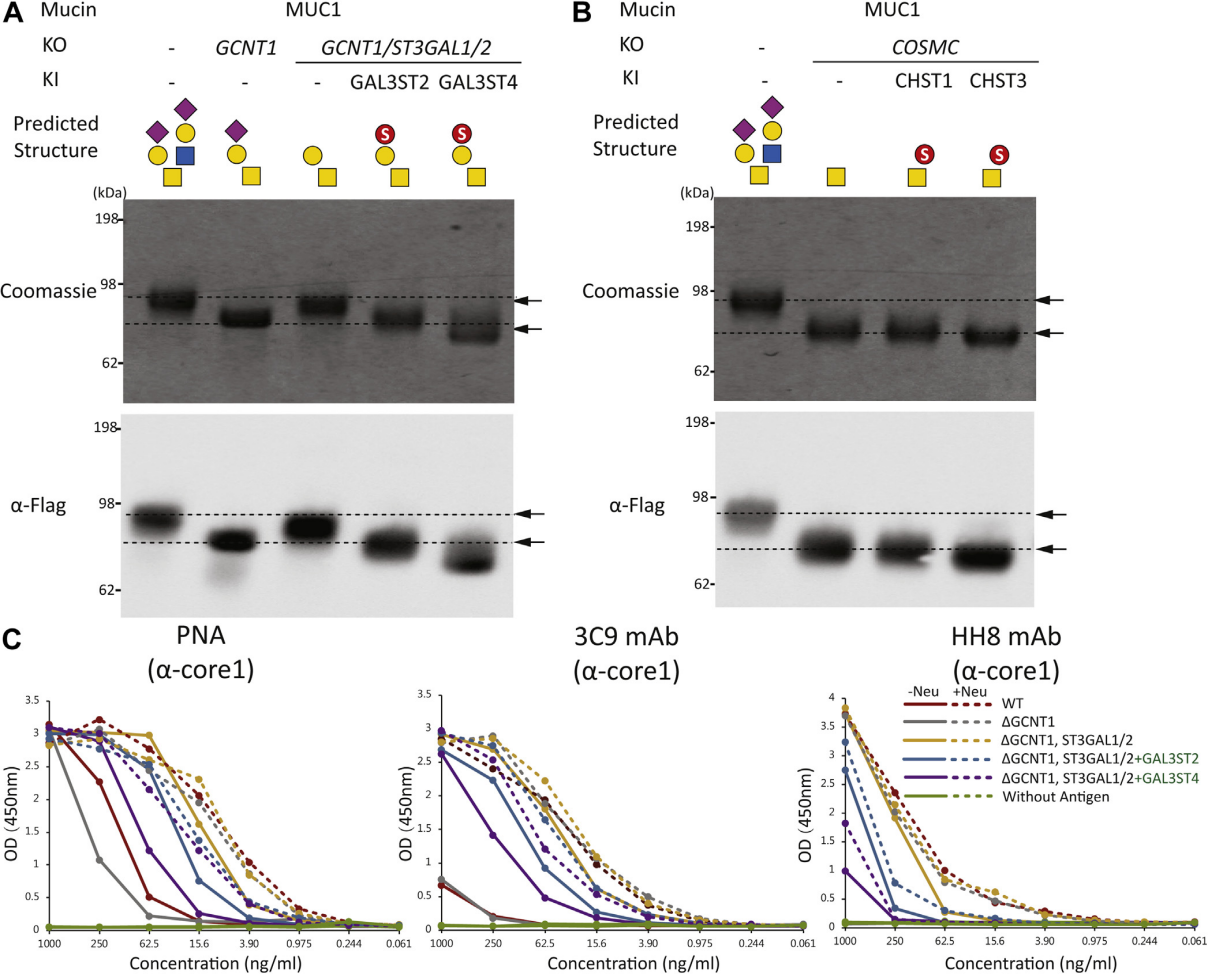
**Characterization of the secreted mucin1 TR reporter with 3-*O*-sulfation of core1.***A* and *B*, SDS-PAGE (Coomassie) and Western blot analysis (anti-FLAG) of the purified mucin1 TR reporter produced in glycoengineered HEK293 cells as indicated with KI of GAL3ST2 and GAL3ST4 for installation of 3-*O*-sulfation on core1 (T) and with KI of CHST1 and CHST3 for installation of 6-*O*-sulfation of Tn. *C*, ELISA analysis with antigen titrations of the glycoengineered-purified mucin1 TR reporters with (*dashed lines*) or without (*solid lines*) neuraminidase pretreatment and probed by PNA lectin, 3C9 and HH8 mAbs. HEK293, human embryonic kidney 293 cell line; KI, knockin; TR, tandem repeat 1.

To further evaluate sulfation capacities on O-glycosylation, we expressed previously designed human-secreted mucin TR O-glycan reporters in the engineered cells (Figs. 1*B* and S5) (38, 40). We first analyzed the purified mucin1 reporter by SDS-PAGE and Western blot analysis and observed clear shifts in mobility when expressed in cells with different O-glycosylation capacities (Fig. 3*A*). Loss of core2 O-glycans (HEK293^KO^ ^*GCNT1*^) enhanced migration, whereas further loss of core1 sialylation (HEK293^KO^ ^*GCNT1*,^ ^*ST3GAL1/2*^) slowed migration. Importantly, KI of GAL3ST2 and GAL3ST4 in the latter cells with O-glycosylation limited to core1 without α2–3 sialylation (HEK293^KO^ ^*GCNT1*,^ ^*ST3GAL1/2*, KI GAL3ST2^ and HEK293^KO^ ^*GCNT1*,^ ^*ST3GAL1/2*, KI GAL3ST4^) reverted the faster migration and for GAL3ST4 even increased the migration further, indicating efficient sulfation. The effect of sialic acids on migration was evaluated by neuraminidase treatment, and the migration changes induced by GAL3ST2 and GAL3ST4 were unaffected (Fig. S6*A*). We further analyzed the mucin1 reporter expressed in HEK293^WT^ cells with endogenous sialylation capacity (HEK293^KI GAL3ST2^ and HEK293^KI GAL3ST4^) and without core2 (HEK293^KO^ ^*GCNT1*, KI GAL3ST2^ and HEK293^KO^ ^*GCNT1*, KI GAL3ST4^) (Fig. S6*B*). KI of GAL3ST2 in the background of core2 O-glycosylation in HEK293^WT^ cells induced a marked shift in migration of the mucin1 reporter, whereas this did not occur in the background of core1 O-glycosylation (HEK293^KO^ ^*GCNT1*, KI GAL3ST2^). This indicates that GAL3ST2 can override 3-*O*-sulfation on the LacNAc disaccharide of the core2 branch. In contrast, KI of GAL3ST4 did not change mobility of the mucin1 reporter in the WT background but produced a slight shift in migration in combination with KO of *GCNT1*, which suggests that GAL3ST4 prefers the core1 substrate (Fig. S6*B*). We also analyzed the purified mucin1 reporters by ELISA with PNA lectin and anti-T mAbs (Fig. 3*C*), which corroborated the findings.

We then analyzed the potential of CHST1 or CHST3 for 6-*O*-sulfation of Tn with the mucin1 reporter expressed in HEK293^KO^ ^*COSMC*^ (Fig. 3*B*). While KI of CHST1 did not appear to affect the migration of the reporter, we did observe a distinct shift toward faster migration with KI of CHST3. This suggested that CHST3 does confer some degree of sulfation of Tn in the absence of competition by sialylation, despite that we could not demonstrate this with flow cytometry analysis relying on loss of anti-Tn reactivity (Fig. S4). It is important though to note that low levels of sulfation capping may not substantially inhibit binding of the anti-Tn reagents.

Finally, we tested the engineering of 3-*O*-sulfation of core1 O-glycans with mucin2 and mucin7 TR reporters (Fig. S7). The reporter design enables facile exchange of mucin TRs (38, 40), and we stably expressed secreted TR reporters for mucin2 or mucin7 in HEK293^KO^ ^*GCNT1, ST3GAL1/2*, KI GAL3ST2^ and HEK293^KO^ ^*GCNT1*,^ ^*ST3GAL1/2*, KI GAL3ST4^ cells. Interestingly, the mucin7 reporter produced the same shift to faster migration as found for the MUC1 reporter, whereas the mucin2 reporter only produced a slight shift suggesting that the mucin2 TR is less prone to core1 sulfation.

### Analysis of the mucin1 TR reporter by MS

The human mucin1 TRs of 20 amino acids contain a conserved PDTR sequence motif enabling endo-AspN digestion and bottom–up MS analysis. The mucin1 reporter comprises 6.5 TRs, each with five potential O-glycosites and a maximum of 34 O-glycosites (Fig. S5). LC–MS/MS analysis of the mucin1 reporter expressed with Tn (HEK293^KO^ ^*COSMC*^) and T (HEK293^KO^ ^*GCNT1*,^ ^*ST3GAL1/2*^) O-glycans showed that all potential O-glycosites were nearly fully occupied with the Thr in PDTR being the most variable site and slight lower occupancy for T compared with Tn O-glycosylation (Fig. S8, *A* and *B*). We observed very low amount of an excess of one HexNAc compared with the total number of Ser/Thr potential O-glycosites, as discussed previously (40). A previous study has predicted the presence of GalNAc–GalNAc O-glycan human meconium (49). Glycosylation of this site is performed by the GalNAc-T4 isoenzyme and requires prior glycosylation at other sites in the mucin1 TR (50). Analysis of the sulfated mucin1 reporters expressed in cells with 3-*O*-sulfation capacities (HEK293^KO^ ^*GCNT1*,^ ^*ST3GAL1/2*, KI GAL3ST2^ and HEK293^KO^ ^*GCNT1*,^ ^*ST3GAL1/2*, KI GAL3ST4^) showed that the predominant mucin1 TR glycoforms were not sulfated, but glycoforms with 1 to 3 sulfate groups on core1 O-glycans per TR were clearly found, and the general O-glycan occupancy was slightly reduced from 4–5 to 3–4 O-glycans per TR (Fig. S8, *C* and *D*). Because of low quality of MS/MS data for sulfated glycopeptides, quantitative analysis of the characteristic precursor ions was performed purely by extracted-ion chromatogram of the theoretical masses corresponding to sulfo-T glycopeptides with various distribution of T and sulfated T epitopes per TR. MS/MS spectra from HCD scans confirmed the existence of the sulfated T on the TR despite the missing information of site localization (Fig. S8*D*), but the quality of the obtained MS/MS spectra did not enable us to identify specific sites of sulfation.

**Figure 4:**
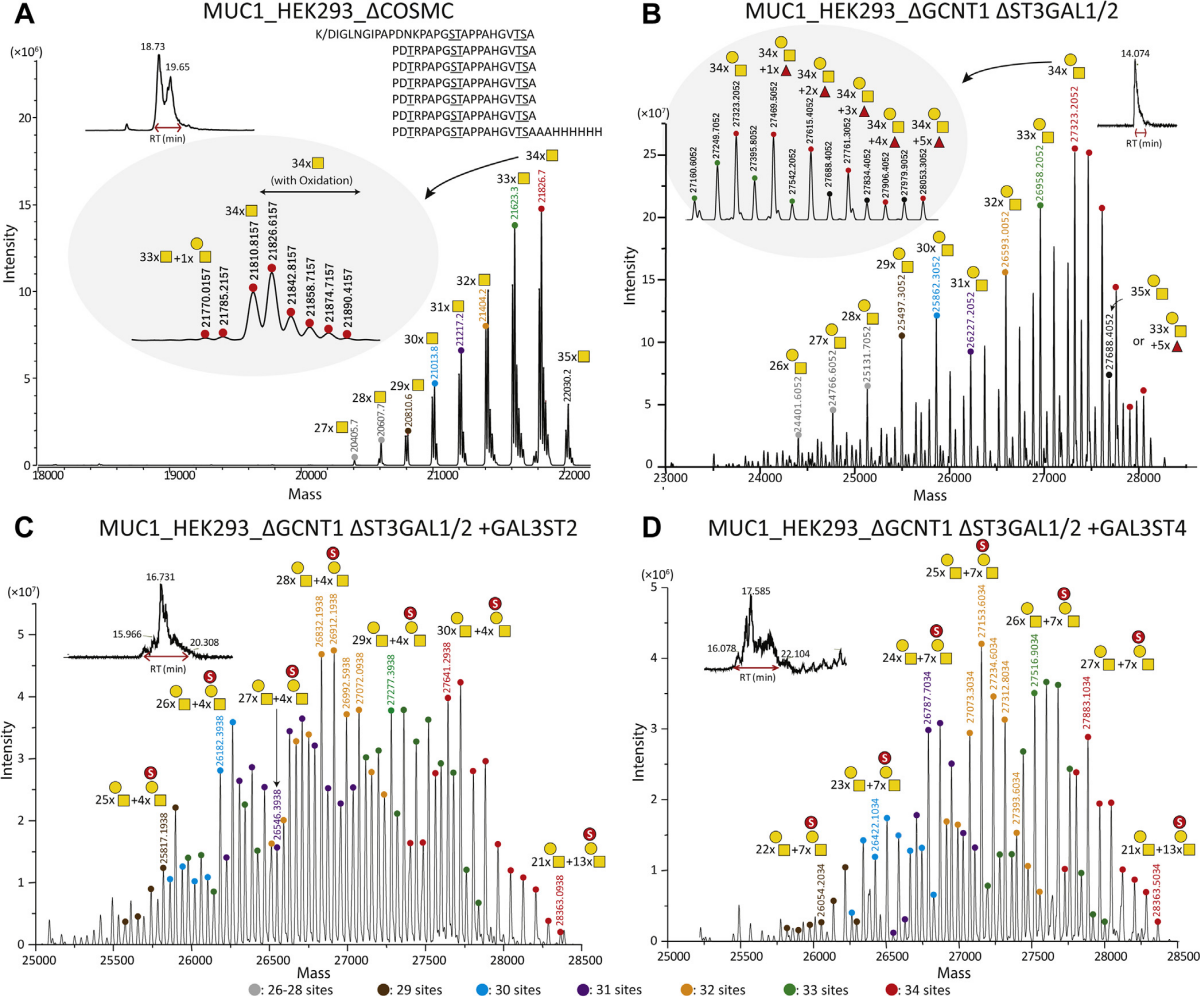
**Intact mass analysis of the Lys-C–purified mucin1 TR O-glycodomain.** Zero-charge deconvoluted intact mass spectra of the C4 HPLC-purified mucin1 TR O-glycodomain derived from reporters expressed in glycoengineered HEK293 cells as indicated with neuraminidase treatment. *A*, the simplest mucin1 O-glycodomain with Tn O-glycans. *B*, the mucin1 O-glycodomain with core1 (T) O-glycans. Note, the emergence of glycoforms containing one predicted Fuc residue, presumably a result of loss of competing α2–3 sialylation. *C*, the mucin1 O-glycodomain of T O-glycans with KI of GAL3ST2. *D*, the mucin1 O-glycodomain of T O-glycans with KI of GAL3ST4. Peaks are assigned *color dot codes* to represent the number of O-glycans as indicated and different numbers of sulfate groups. *Gray boxes* in *A* and *B* show examples of satellite peaks zoomed in. Independent experiments were performed at least three times with similar results. HEK293, human embryonic kidney 293 cell line; TR, tandem repeat.

To further address stoichiometry of the O-glycosylation, we released the TR O-glycodomain from the GFP-mucin1 reporter by Lys-C digestion and performed intact MS analysis of the C4 HPLC-isolated domain (Fig. 4*A*). It was determined that the mucin1 TRs are highly occupied with Tn as well as T with the predominant identified glycoforms being those with 33 to 34 HexNAc or Hex-HexNAc residues and thus not reflecting the slightly lower occupancy observed with the T glycoform by the bottom–up analysis (Fig. S8*B*). Note that samples were pretreated with neuraminidase to remove potential α2–6 sialic acids to the internal GalNAc residue. Next, we analyzed the sulfated mucin1 reporter expressed in cells with 3-*O*-sulfation capacities (HEK293^KO^ ^*GCNT1*,^ ^*ST3GAL1/2*, KI GAL3ST2^ and HEK293^KO^ ^*GCNT1*,^ ^*ST3GAL1/2*, KI GAL3ST4^), which confirmed that sulfation was incomplete with the predominant glycoforms predicted to have 24 to 30 core1 O-glycans and 4 to 7 sulfated core1 O-glycans (Fig. 4, *C* and *D*). The most sulfated detectable proteoform for both GAL3ST2 and GAL3ST4 KI was 21 T and 13 sulfate groups.

Finally, despite the finding that mucin1 expressed in HEK293^KO^ ^*COSMC*, KI CHST3^ resulted a slight change in SDS-PAGE migration (Fig. 3*B*), we were unable to identify evidence of sulfation in the intact mass and bottom–up analysis (data not shown).

### Binding specificity of GAL-4

**Figure 5:**
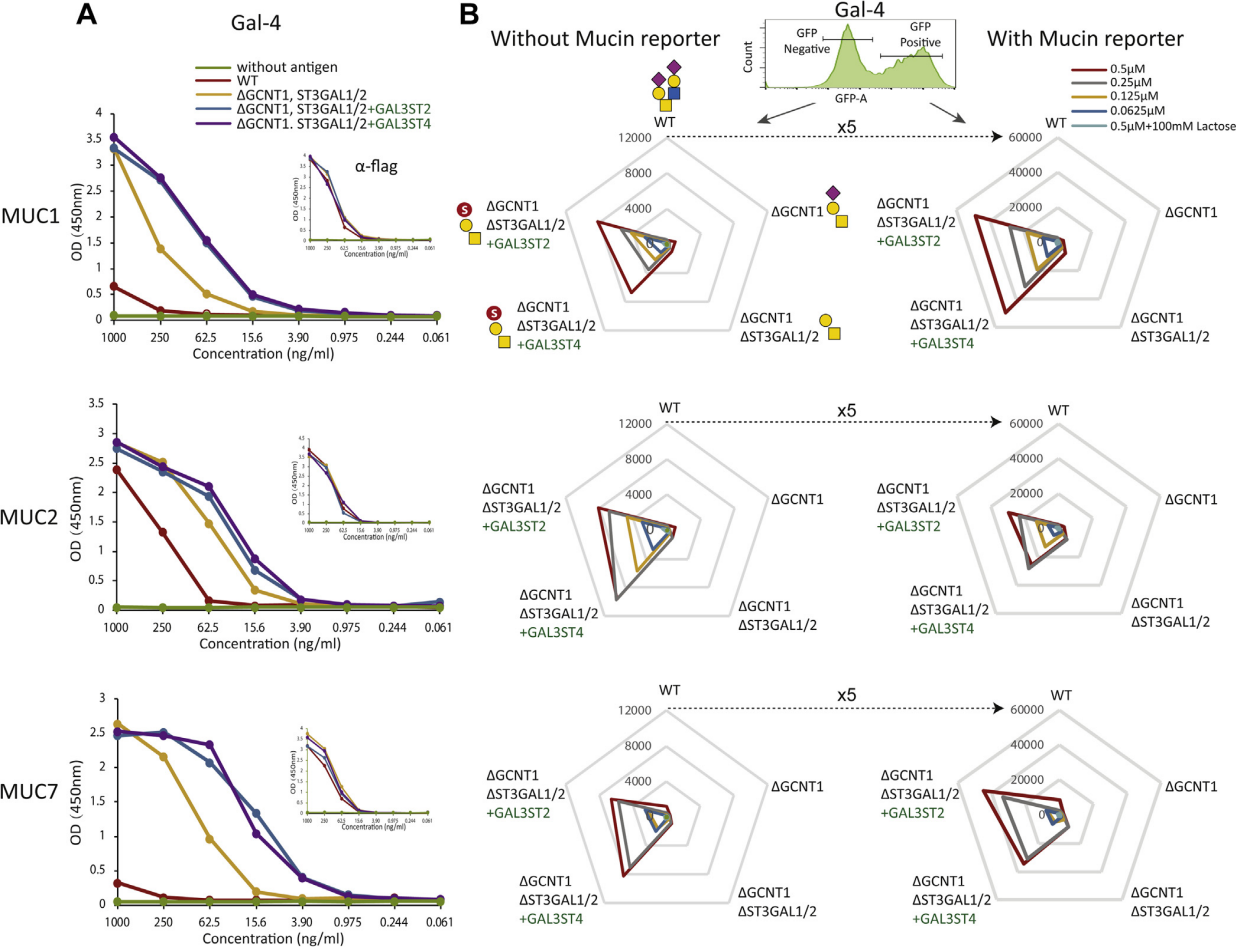
**Analysis of binding specificity of GAL-4.***A*, ELISA analysis with purified glycoengineered mucin (mucin1, mucin2, and mucin7) TR reporters (antigen titration from 1 μg/ml) by detecting with GAL-4 and anti-FLAG. *B*, flow cytometry analysis of transiently expressed membrane-bound human TR reporters in glycoengineered cells as indicated. *Radar charts* show MFIs of Gal-4 binding measured by gating for the GFP-positive (transfected and mucin TR expressing, *solid line*) and GFP-negative (nontransfected and not expressing, *dashed line*) cell populations. Independent experiments were performed at least three times with similar results. GAL-4, galectin-4; TR, tandem repeat.

GAL-4 preferentially binds 3-*O*-sulfated Gal residues and was reported to bind 3-*O*-sulfated core1 O-glycans (51, 52). We therefore used GAL-4 to probe the engineering of sulfation. We first tested GAL-4 binding by ELISA with secreted purified mucin1 (34 O-glycosites), mucin2 (86 O-glycosites), and mucin7 (66 O-glycosites) TR reporters. GAL-4 did not bind significantly to reporters expressed in HEK293^WT^, with the exception of weak binding to the mucin2 reporter with highest density of O-glycans (Fig. 5*A*). GAL-4 bound to all reporters when expressed in HEK293^KO^ ^*GCNT1*,^ ^*ST3GAL1/2*^ cells without α2–3 sialylation of core1 O-glycans, and the binding was markedly enhanced by introduction of 3-*O*-sulfation (HEK293^KO^ ^*GCNT1*,^ ^*ST3GAL1/2*, KI GAL3ST2^ and HEK293^KO^ ^*GCNT1*,^ ^*ST3GAL1/2*, KI GAL3ST2^ cells) (Fig. 5*A*). We also tested this by the cell-based display platform using transient expression of equivalent membrane-bound TR reporters for mucin1, mucin2, and mucin7 in engineered cells (40). GAL-4 binding was analyzed by gating for GFP-positive (transfected) and GFP-negative (nontransfected) cell populations, and GAL-4 did not bind or bound only weakly to HEK293^WT^, HEK293^KO^ ^*GCNT1*^, and HEK293^KO^ ^*GCNT1*,^ ^*ST3GAL1/2*^ cells with and without expression of the mucin reporters. However, binding was significantly enhanced in HEK293^KO^ ^*GCNT1*,^ ^*ST3GAL1/2*, KI GAL3ST2^ and HEK293^KO^ ^*GCNT1*,^ ^*ST3GAL1/2*, KI GAL3ST4^ cells, and expression of the mucin reporters provided substantial higher binding (approximately five times). Here, again the MUC2 reporter produced lower binding than the mucin1 and mucin7 reporters (Fig. 5*B*).

## Discussion

The mammalian cell lines, HEK293 and Chinese hamster ovary, widely used for recombinant expression of glycoproteins do not have sulfation capacities for N-glycoproteins and O-glycoproteins and therefore offer nascent systems to reconstruct sulfation of different types of glycans. The immature truncated T, Tn, and STn O-glycans represent prominent cancer-associated glycosylation features found only on O-glycoproteins, including mucins and mucin-like glycoproteins (19), and capping of these by sulfation has been reported (36, 37). Here, we aimed at shedding light on the genetic and biosynthetic regulation of sulfation of these cancer-associated O-glycans, and we were able to establish stable engineered HEK293 cells with 3-*O*-sulfation capacities. Introduction of GAL3ST4 provided selective 3-*O*-sulfation of core1 O-glycans (Galβ1–3GalNAc), whereas introduction of GAL3ST2 provided broader 3-*O*-sulfation of core1 and core2 O-glycans (Galβ1–3GalNAc and Galβ1–4GlcNAc) as well as for glycolipids and N-glycans. We were unable to convincingly demonstrate efficient 6-*O*-sulfation capacity for the truncated Tn O-glycan by introduction of CHST1 or CHST3, but CHST3 remains a candidate since KI of this gene induced a shift in migration of the Tn-mucin1 reporter (Fig. 3*B*).

We used prior knowledge of sulfotransferases mainly derived from *in vitro* enzyme assays and phylogenic analysis to identify the most likely candidate sulfotransferases for sulfation of the T and Tn cancer-associated O-glycans (Fig. S1). The putative sulfotransferases involved in the synthesis of 3-*O*-Sulfo-T and 6-*O*-Sulfo-Tn O-glycans were predicted to be found among the 3-*O*-sulfotransferase isoenzymes, GAL3ST2 and GAL3ST4, and the 6-*O*-sulfotransferase isoenzymes, CHST1 and CHST3, respectively (Fig. S1) (36, 43, 44). The engineering strategies included KO of the endogenous O-glycosylation capacities (core1 O-glycan extension [KO *COSMC*], branching [KO *GCNT1*], and sialylation [KO *ST3GAL1/2*]) that potentially outcompete sulfation. We may not have identified and studied the most optimal enzymes. The 3-*O*-sulfation of core1 by GAL3ST2 and GAL3ST4 was only demonstrated convincingly in the absence of α2–3 sialylation (Fig. S6, *A* and *B*), and the sulfation with these was incomplete with most T O-glycans unmodified despite lack of competition for the T-acceptor substrate as evaluated by intact and bottom–up MS analysis (Figs. 4 and S8). Interestingly though, the immunochemical analysis using flow cytometry (Fig. 2) and ELISA (Fig. 3) indicated that sulfation by GAL3ST4, and to lesser extent GAL3ST2, substantially reduced binding with anti-T reagents. This was particularly evident not only with the mAbs HH8 and 3C9 but also with the more sensitive PNA lectin. Moreover, KI of the GAL3ST sulfotransferases produced clear shifts in migration of the rather distinct mucin1 reporter bands by SDS-PAGE analysis, and with GAL3ST2, the band comigrated with the sialylated glycoforms, whereas with GAL3ST4, the band migrated even faster than this (Figs. 3*A* and S6). Structural analysis of sulfated glycopeptides by LS–MS/MS is challenging. Enrichment of glycopeptides is needed, and sulfated glycans pose particular challenges because of poor ionization and instability of sulfate groups during the MS/MS fragmentation process. Stabilization of SO_3_ group by ion-paring reagent is a method of choice for identification of glycosites in sulfated glycopeptides, and the use of 3K (Lys–Lys–Lys) ion pair complexes has improved analysis of sulfated N-glycopeptides (53). Recent improvements in analysis in negative ion mode after sialic acid neutralization have advanced analysis of sulfated glycan (8, 10), and use of the negative ion mode is a promising approach for analysis of sulfoglycopeptides (54, 55). Here, we were unable to further define degree and position of the added sulfation introduced, but our study suggests that 3-*O*-sulfation of at least for core1 O-glycans may be an inefficient process compared with sialylation.

Our interest in enabling the production of core1 (T) and Tn O-glycans with sulfate partly stems from a hypothesis that these glycoforms may represent important tumor-associated epitopes that have hereto been overlooked partly because of analytic constraints and partly because of lack of appropriate antibodies and binding proteins. The T, Tn, and STn O-glycan epitopes constitute the most prevalent cancer-associated glycan epitopes (56, 57), and 3-*O*-sulfation of the Gal and 6-*O*-sulfation of both Gal and GalNAc in the core1 O-glycan have been identified on mucin1 purified from a breast cancer cell line, as well as on mucin7 isolated from arthritis patients (15, 36, 37). We previously demonstrated how availability of cells with homogeneous O-glycoforms allow for the production of immunogens that can be used to elicit novel antibodies with highly select specificity for aberrant O-glycoforms of glycoproteins (58), and we envisage that the developed cell expression system for sulfated core1 O-glycans can be used to develop such probes.

We were unable to convincingly demonstrate sulfation of Tn, although the SDS-PAGE analysis of the mucin1 reporter expressed in cells with CHST3 and not CHST1 did suggest some degree of sulfation (Fig. 3*B*). CHST1 was initially found to 6-*O*-sulfate the Gal residue in KS and later reported to have activity with mSTa but relatively lower or no activity toward T and Tn structures by *in vitro* assays (36, 45). More recently, CHST1 was shown to induce strong binding of Siglec-7 and 15 to core1 O-glycans (25, 36, 45). None of the 6-*O*-sulfotransferases have been reported to sulfate GalNAc in Tn structure, whereas CHST1 is reported to sulfate GalNAc in mSTa, and CHST3 is evolutionary the most closest one to CHST1, which can sulfate GalNAc residue in chondroitin sulfate (36, 46, 59) (Fig. S1).

Engineering sulfation capacities in HEK293 cells provides a valuable platform for cell-based display of sulfated glycans and interrogation of the specificities of glycan-binding proteins (25, 38, 60, 61). Glycoengineering in mammalian cells has mainly focused on sialylation, and only few studies have addressed sulfation (13, 62). We used the cell-based glycan display platform combined with expression of mucin reporters to confirm the binding specificity of GAL-4 for sulfated O-glycans. GAL-4 was previously shown to bind core1 O-glycans with strong preference for 3-*O*-sulfated O-glycans (51, 52), and this was fully recapitulated with the display platform where GAL-4 binding to WT and core1 engineered HEK293 cells was negligible or weak, respectively, whereas KI of GAL3ST2 and GAL3ST4 induced strong binding that was further amplified by expression of mucin reporters (Fig. 5). Recently, we used the same cell engineering strategy and discovered natural ligands related to sulfation for several Siglecs (Siglec-3, Siglec-7, Siglec-8, and Siglec-15) that required expression of the CHST1 6-*O*-sulfotransferase (25). We were unable to demonstrate clear functions for CHST1 as well as CHST3 in 6-*O*-sulfation of the cancer-associated Tn O-glycan. CHST1 is the key player for the synthesis of KS, which is the ligand of Siglec-8 and predicted to primarily 6-*O*-sulfate the galactose residue in LacNAc (Galβ1–4GlcNAc) and α2–3 sialylated LacNAc (63, 64, 65). Moreover, CHST1 was reported to have activity on GalNAc residues with mSTa but has relatively lower or no activity toward T or Tn O-glycans *in vitro* (36), and recently, CHST1 was found to affect core1 O-glycosylation and Siglec-7/15 binding (25). CHST3 was the other likely candidate for synthesis of 6-*O*-sulfated Tn regarding its known role in 6-*O*-sulfation of GalNAc residues (46, 59).

We simplified the complexity of glycosylation by KO of the core2 synthase (GCNT1) and sialyltransferase (ST3GAL1/2), which resulted in production of highly homogeneous molecules and further ensured by neuraminidase treatment of isolated reporters before MS analysis. The mucin1 TR reporters with simplified (Tn and T) and homogenous O-glycans enabled intact mass analysis of the released O-glycodomain (Fig. 4), which provided comparable results for bottom–up analysis (Fig. S8). While intact MS analysis is a powerful tool to explore the glycoproteoform distribution (66), it is clearly still a challenge to apply this for analysis of the inherent microheterogeneity of glycoproteins (67). Combining intact MS and bottom–up LC–MS/MS analysis is often necessary to deduce glycostructures and glycosites in full. Although the MS/MS spectra quality did not provide confident evidence to identify sulfoglycopeptides, HCD MS/MS spectra showed the presence of sulfo-T oxonium fragment ions in the mucin1 TRs (Fig. S8*D*, *inset*). A combination of the intact mass analysis of the full mucin1 TR construct together with the extracted ion chromatogram for the predicted sulfated glycopeptide masses from corresponding bottom–up data suggested that the most abundant glycoproteoform was fully occupied by the core1/T structure with one sulfate group for at least four of seven TRs with KI of GAL3ST2 (Figs. 4*C* and S8*C*). The most abundant glycoproteoform with KI of GAL3ST4 was predicted to be with at least one sulfo-T residue per TR (Fig. 4*D*). For both samples, the highest glycoproteoforms detected were with up to 21 T and 13 sulfo-T epitopes, which would correspond to at least two sulfate groups per TR. We were unable to identify sulfation of the Tn O-glycans on the mucin1 reporter expressed in cells with KI of CHST3 despite the clear shift in SDS-PAGE migration, and further advancements are clearly needed.

In summary, we have developed a sustainable cell-based platform for display and production of mucins with sulfated O-glycans. The strategy can be expanded to other sulfotransferases and with focus on more complex O-glycans or other glycoconjugates.

## Experimental procedures

### Gene targeting in HEK293 cells

HEK293 cells were cultured in Dulbecco's modified Eagle's medium (Sigma) media supplemented with 10% fetal bovine serum (Sigma) and 4 mM GlutaMAX (Gibco) at 37 °C and 5% CO_2_. Gene targeting was performed by the CRISPR/Cas9 KO with a validated gRNA library for all human glycosyltransferases (GlycoCRISPR) and the site-directed KI using a modified ObLiGaRe-targeted KI strategy as previously described (41, 68). Briefly, HEK293 cells were seeded in 6-well plates 1 day prior the transfection and cotransfected by using Lipofectamine 3000 with 1 μg of gRNA and 1 μg of GFP-tagged Cas9-PBKS for CRISPR/Cas9 KO or with 1.5 μg of each ZFN tagged with GFP/crimson and 3.5 μg donor plasmid for targeted KI. Cells were harvested 24 h after transfection, and the GFP-labeled bulk pool of cells were enriched by fluorescence-activated cell sorting (SH800; SONY). After 1 week in culture, cells were single sorted into 96-well plates. KI clones were screened by the junction PCR with primers specific for the junction area between the donor plasmid and the AAVS1 locus, and a primer set flanking the targeted KI locus was used to characterize the allelic insertion status. All KO clones were screened by the indel profile of target genes by Indel Detection by Amplicon Analysis (69), and selected clones were further verified by Sanger sequencing.

### NHS-biotin labeling of GAL-4

Recombinant intact human GAL-4 (amino acids 1–323; NP_006140.1) without added tags was produced from vector pET28c in *Escherichia coli* BL21 Star (DE3) cells (Invitrogen) and then purified by affinity chromatography on lactosyl-sepharose as previously described for galectin-3 (70). A 20-fold molar excess of NHS-Biotin (Thermo Fisher Scientific) dissolved in dimethyl sulfoxide was added to a 2 mg/ml of GAL-4 solution in PBS buffer. After 2 h incubation on ice, labeled Gal-4 was separated from the unreacted dye by a buffer change to PBS on a PD10 column.

### Cell binding assays

Cell binding assays were performed on ice with mAbs, biotinylated-lectins and biotinylated GAL-4. Optionally, cells were treated with 150 mU/ml *Clostridium perfringens* neuraminidase (Sigma) for 1 h at 37 °C. All mouse mAbs, 3C9, HH8, 5F4, and NUH2 (71) and biotinylated PNA and vicia villosa agglutinin lectins (Vector Laboratories) were incubated at different concentrations for 1 h, followed by washing and incubating with Alexa Fluor 488–conjugated rabbit antimouse IgG/IgM (Thermo Fisher Scientific) or Alexa Fluor 488–conjugated streptavidin (Thermo Fisher Scientific) for 1 h. To assess the GAL-4 binding, cells were prewashed in 100 mM lactose to remove the endogenous binding and incubated with biotinylated GAL-4 on ice for 30 min followed by incubation with Alexa Fluor 647–conjugated streptavidin for 30 min (Thermo Fisher Scientific). Washing was performed with PBS–bovine serum albumin (BSA) (1× PBS containing 1% BSA [Sigma–Aldrich]), and cells were resuspended for flow cytometry analysis (SA3800; SONY).

### Expression and purification of human mucin reporter proteins

Secreted and transmembrane human mucin TR reporters for mucin1, mucin2, and mucin7 were designed and synthesized as previously reported (Fig. 1*B*) (38, 40). HEK293 cells were transfected with the secreted mucin TR reporters, and stable pools were established by G418 selection. After the enrichment of the GFP-positive population by fluorescence-activated cell sorting (SH800; SONY), cells were seeded at a cell density of 5 × 10^5^ cells/ml in 200 ml of serum-free F17 culture media (Thermo Fisher Scientific) supplemented with 0.1% Kolliphor P188 (Sigma) and 4 mM GlutaMax and cultured at 37 °C and 5% CO_2_ under constant agitation (120 rpm). After 4 to 5 days of culture, the supernatant was harvested and purified by nickel affinity chromatography. Media were mixed 3:1 (v/v) in 4× binding buffer (100 mM sodium phosphate, pH 7.4, and 2 M NaCl) and applied to self-packed nickel–nitrilotriacetic acid affinity resin column (Qiagen), pre-equilibrated in washing buffer (25 mM sodium phosphate, pH 7.4, 500 mM NaCl, and 20 mM imidazole). After washing, bound protein was eluted with 200 mM imidazole in washing buffer, analyzed by SDS-PAGE with Coomassie staining, and desalted by PD-10 column (GE Healthcare). Purified mucins were quantified by BCA Protein Assay Kit (Thermo Fisher Scientific). Western blot analysis was performed using NuPAGE Bis–Tris 4 to 12% gels (Thermo Fisher Scientific) with transfer to nitrocellulose membranes (0.45 μm; Bio-Rad) for 60 min at 100 V constant. Membranes were blocked with 5% skimmed milk in Tris-buffered saline with Tween-20 for 1 h at room temperature (RT), incubated at 4 °C overnight with 0.2 μg/ml anti-FLAG M2-Peroxidase-HRP–conjugated mAb (Sigma) diluted in 5% skimmed milk in Tris-buffered saline with Tween-20, and developed with Pierce ECL substrate (Thermo Fisher Scientific) and visualized on BioSpectrum (UVP BioImaging Systems).

### ELISA

MaxiSorp 96-well plates (Nunc) were coated overnight at 4 °C in carbonate–bicarbonate buffer (pH 9.6) and blocked in PLI-P (PO_4_^3−^, Na^+^/K^+^, 1% Triton, and 1% BSA) buffer at pH 7.4 for 1 h at RT. Neuraminidase treatment was performed with 50 mU neuraminidase in 50 mM sodium acetate buffer (pH 5.5) for 1 h at 37 °C. Plates were incubated with mAbs (undiluted culture supernatants), biotinylated PNA lectin (200 ng/ml) (Vector Laboratories), or biotinylated GAL-4 (0.5 μM) for 1 h at RT and followed by washing and incubation with HRP-conjugated antimouse Ig (Dako) or streptavidin–HRP (Dako) for 1 h at RT. Development was started by addition with 3,3′,5,5′-Tetramethylbenzidine substrate (Dako) and stopped with 0.5 M H_2_SO_4_, and absorbance read at 450 nm after 5 min.

### Enzyme digestion of purified mucin1 reporter

Approximately 50 μg of purified mucin1 TR reporter (after desalted by PD-10 column) was digested with Lys-C for intact mass analysis or endo-AspN (Roche Applied Science) for bottom–up analysis at 1:50 enzyme:substrate ratio for 16 h at 37 °C with shaking. Lys-C digestion was performed in 50 mM Ambic (pH 7.4) buffer, and endo-AspN digestion was performed in 100 mM Tris–HCl (pH 8.0) buffer. The O-glycodomain of the Lys-C–digested mucin1 reporter was isolated by C4 HPLC (Jupiter, 5 μm, 300 Å, column 250 × 4.6 mm; Phenomenex), using 0.1% TFA and a gradient of 10 to 100% acetonitrile. The endo-AspN–digested mucin1 20-mer TRs were isolated by C18 HPLC (Kinetex; 2.6 μm, 100 Å, column 100 × 2.1 mm; Phenomenex) using 0.1% TFA and a gradient of 10 to 100% acetonitrile. Isolated fractions were dried in freeze drier and resolubilized in water and analyzed by MS.

### Intact mass analysis

Samples were analyzed by EASY-nLC 1200 UHPLC (Thermo Fisher Scientific) interfaced *via* nanoSpray Flex ion source to an on Orbitrap Fusion/Lumos instrument (Thermo Fisher Scientific) using “high” mass range setting in *m/z* range 700 to 4000. Instrument was operated in “low pressure” mode to provide optimal detection of intact protein masses. MS parameter settings: spray voltage 2.2 kV, source fragmentation energy 35 V. All ions were detected in Orbitrap at the resolution of 7500 (at *m/z* 200). The number of microscans was set to 20. The nLC was operated in a single analytical column set up using PicoFrit Emitters (New Objectives; 75 mm inner diameter) packed in-house with C4 phase (Dr Maisch; particle size of 3.0 μm, column length of 16–20 cm). Each sample was injected onto the column and eluted in gradients from 5 to 30% B in 25 min, from 30 to 100% B in 20 min and 100% B for 15 min at 300 nl/min (solvent A, 100% H_2_O; solvent B, 80% acetonitrile; both containing 0.1% [v/v] formic acid).

### LC MS/MS bottom–up analysis

LC MS/MS bottom–up glycopeptide analysis was performed on EASY-nLC 1200 UHPLC (Thermo Fisher Scientific) interfaced *via* nanoSpray Flex ion source to an Orbitrap Fusion/Lumos MS (Thermo Fisher Scientific). Briefly, the nLC was operated in a single analytical column set up using PicoFrit Emitters (New Objectives; 75 mm inner diameter) packed in-house with Reprosil-Pure-AQ C18 phase (Dr Maisch; particle size of 1.9 μm, column length of 19–21 cm). Each sample was injected into the column and eluted in gradients from 3 to 32% B for glycopeptides and 10 to 40% for released and labeled glycans in 45 min at 200 nl/min (solvent A, 100% H_2_O; solvent B, 80% acetonitrile; both containing 0.1% [v/v] formic acid). A precursor MS1 scan (*m/z* 350–2000) of intact peptides was acquired in the Orbitrap at the nominal resolution setting of 120,000, followed by Orbitrap HCD-MS2 and at the nominal resolution setting of 60,000 of the five most abundant multiply charged precursors in the MS1 spectrum; a minimum MS1 signal threshold of 50,000 was used for triggering data-dependent fragmentation events. Targeted MS/MS analysis was performed by setting up a targeted MSn Scan Properties pane.

### MS data analysis

Glycan and glycopeptide compositional analysis was performed from *m/z* features extracted from LC–MS data using in-house written SysBioWare software (72). For *m/z* feature recognition from full MS scans, Minora Feature Detector Node of the Proteome discoverer 2.2 (Thermo Fisher Scientific) was used. The list of precursor ions (*m/z*, charge, peak area) was imported as ASCII data into SysBioWare, and compositional assignment within 3 ppm mass tolerance was performed. The main building blocks used for the compositional analysis were theoretical monoisotopic mass increment of NeuAc, Hex, HexNAc, dHex, and the theoretical monoisotopic mass increment of the most prominent peptide corresponding to each potential glycosites. To generate the potential glycopeptide list, all the glycoforms with an abundance higher than 5% of the most abundant glycoform were used for glycan feature analysis. Raw spectra for intact mass analysis were deconvoluted to zero charge by BioPharma Finder Software (Thermo Fisher Scientific) using default settings. Glycoproteoforms were annotated by in-house written SysBioWare software (72) using average masses of hexose, *N*-acetylhexosamine, and the known backbone mass of mucin1 TR reporter increment.

## Data availability

All data generated or analyzed during this study are included in this article and supporting information files. The MS proteomics data have been deposited to the ProteomeXchange Consortium *via* the PRIDE (73) partner repository with the dataset identifier PXD028982.

## Supporting information

This article contains supporting information (5, 19, 36, 38, 42, 43, 44, 45, 46, 60, 74, 75, 76, 77, 78, 79, 80, 81, 82, 83, 84, 85, 86, 87, 88, 89, 90, 91, 92, 93, 94, 95, 96, 97, 98, 99, 100, 101, 102, 103, 104, 105, 106, 107, 108, 109, 110, 111, 112).

## Conflict of interest

University of Copenhagen has filed a patent application on the cell-based display platform. GlycoDisplay ApS, Copenhagen, Denmark, has obtained a license to the field of the patent application. Y. N. and H. C. are cofounders of GlycoDisplay ApS and hold ownerships in the company. H. L. is cofounder and consultant with Galecto, Inc. All other authors declare that they have no conflicts of interest with the contents of this article.

## References

[1] Sulkin N.M. (1955). Histochemical studies on mucoproteins in nerve cells of the dog. J. Biophys. Biochem. Cytol..

[2] Jin C., Kenny D.T., Skoog E.C., Padra M., Adamczyk B., Vitizeva V., Thorell A., Venkatakrishnan V., Linden S.K., Karlsson N.G. (2017). Structural diversity of human gastric mucin glycans. Mol. Cell. Proteomics.

[3] Xia B., Royall J.A., Damera G., Sachdev G.P., Cummings R.D. (2005). Altered O-glycosylation and sulfation of airway mucins associated with cystic fibrosis. Glycobiology.

[4] Joncquel Chevalier Curt M., Lecointe K., Mihalache A., Rossez Y., Gosset P., Leonard R., Robbe-Masselot C. (2015). Alteration or adaptation, the two roads for human gastric mucin glycosylation infected by Helicobacter pylori. Glycobiology.

[5] Habuchi O., Suzuki Y., Fukuta M. (1997). Sulfation of sialyl lactosamine oligosaccharides by chondroitin 6-sulfotransferase. Glycobiology.

[6] Derry C.J., Faveeuw C., Mordsley K.R., Ager A. (1999). Novel chondroitin sulfate-modified ligands for L-selectin on lymph node high endothelial venules. Eur. J. Immunol..

[7] Yu S.Y., Wu S.W., Hsiao H.H., Khoo K.H. (2009). Enabling techniques and strategic workflow for sulfoglycomics based on mass spectrometry mapping and sequencing of permethylated sulfated glycans. Glycobiology.

[8] Khoo K.H., Yu S.Y. (2010). Mass spectrometric analysis of sulfated N- and O-glycans. Methods Enzymol..

[9] Chen J.Y., Huang H.H., Yu S.Y., Wu S.J., Kannagi R., Khoo K.H. (2018). Concerted mass spectrometry-based glycomic approach for precision mapping of sulfo sialylated N-glycans on human peripheral blood mononuclear cells and lymphocytes. Glycobiology.

[10] Cheng C.-W., Chou C.-C., Hsieh H.-W., Tu Z., Lin C.-H., Nycholat C., Fukuda M., Khoo K.-H. (2015). Efficient mapping of sulfated glycotopes by negative ion mode nanoLC–MS/MS-based sulfoglycomic analysis of permethylated glycans. Anal. Chem..

[11] Cheng P.F., Snovida S., Ho M.Y., Cheng C.W., Wu A.M., Khoo K.H. (2013). Increasing the depth of mass spectrometry-based glycomic coverage by additional dimensions of sulfoglycomics and target analysis of permethylated glycans. Anal. Bioanal. Chem..

[12] Khoo K.-H. (2015). Glycoscience: Biology and Medicine.

[13] Yu S.Y., Hsiao C.T., Izawa M., Yusa A., Ishida H., Nakamura S., Yagi H., Kannagi R., Khoo K.H. (2018). Distinct substrate specificities of human GlcNAc-6-sulfotransferases revealed by mass spectrometry-based sulfoglycomic analysis. J. Biol. Chem..

[14] Issa S.M.A., Vitiazeva V., Hayes C.A., Karlsson N.G. (2018). Higher energy collisional dissociation mass spectrometry of sulfated O-linked oligosaccharides. J. Proteome Res..

[15] Flowers S.A., Lane C.S., Karlsson N.G. (2019). Deciphering isomers with a multiple reaction monitoring method for the complete detectable O-glycan repertoire of the candidate therapeutic, lubricin. Anal. Chem..

[16] Karlsson N.G., Thomsson K.A. (2009). Salivary MUC7 is a major carrier of blood group I type O-linked oligosaccharides serving as the scaffold for sialyl Lewis x. Glycobiology.

[17] Steentoft C., Vakhrushev S.Y., Joshi H.J., Kong Y., Vester-Christensen M.B., Schjoldager K.T., Lavrsen K., Dabelsteen S., Pedersen N.B., Marcos-Silva L., Gupta R., Bennett E.P., Mandel U., Brunak S., Wandall H.H. (2013). Precision mapping of the human O-GalNAc glycoproteome through SimpleCell technology. EMBO J..

[18] Brockhausen I., Stanley P., Varki A., Cummings R.D., Esko J.D., Stanley P., Hart G.W., Aebi M., Darvill A.G., Kinoshita T., Packer N.H., Prestegard J.H., Schnaar R.L., Seeberger P.H. (2015).

[19] Schjoldager K.T., Narimatsu Y., Joshi H.J., Clausen H. (2020). Global view of human protein glycosylation pathways and functions. Nat. Rev. Mol. Cell Biol..

[20] Duan S., Paulson J.C. (2020). Siglecs as immune cell checkpoints in disease. Annu. Rev. Immunol..

[21] Kawashima H., Petryniak B., Hiraoka N., Mitoma J., Huckaby V., Nakayama J., Uchimura K., Kadomatsu K., Muramatsu T., Lowe J.B., Fukuda M. (2005). N-acetylglucosamine-6-O-sulfotransferases 1 and 2 cooperatively control lymphocyte homing through L-selectin ligand biosynthesis in high endothelial venules. Nat. Immunol..

[22] Bum-Erdene K., Leffler H., Nilsson U.J., Blanchard H. (2016). Structural characterisation of human galectin-4 N-terminal carbohydrate recognition domain in complex with glycerol, lactose, 3'-sulfo-lactose, and 2'-fucosyllactose. Sci. Rep..

[23] Rosen S.D. (2004). Ligands for L-selectin: Homing, inflammation, and beyond. Annu. Rev. Immunol..

[24] Macauley M.S., Kawasaki N., Peng W., Wang S.H., He Y., Arlian B.M., McBride R., Kannagi R., Khoo K.H., Paulson J.C. (2015). Unmasking of CD22 co-receptor on germinal center B-cells occurs by alternative mechanisms in mouse and man. J. Biol. Chem..

[25] Bull C., Nason R., Sun L., Van Coillie J., Madriz Sorensen D., Moons S.J., Yang Z., Arbitman S., Fernandes S.M., Furukawa S., McBride R., Nycholat C.M., Adema G.J., Paulson J.C., Schnaar R.L. (2021). Probing the binding specificities of human Siglecs by cell-based glycan arrays. Proc. Natl. Acad. Sci. U. S. A..

[26] Streeter P.R., Rouse B.T., Butcher E.C. (1988). Immunohistologic and functional characterization of a vascular addressin involved in lymphocyte homing into peripheral lymph nodes. J. Cell Biol..

[27] Yeh J.C., Hiraoka N., Petryniak B., Nakayama J., Ellies L.G., Rabuka D., Hindsgaul O., Marth J.D., Lowe J.B., Fukuda M. (2001). Novel sulfated lymphocyte homing receptors and their control by a Core1 extension beta 1,3-N-acetylglucosaminyltransferase. Cell.

[28] Chou K.H., Ilyas A.A., Evans J.E., Quarles R.H., Jungalwala F.B. (1985). Structure of a glycolipid reacting with monoclonal IgM in neuropathy and with HNK-1. Biochem. Biophys. Res. Commun..

[29] Ariga T., Kohriyama T., Freddo L., Latov N., Saito M., Kon K., Ando S., Suzuki M., Hemling M.E., Rinehart K.L., Kusunoki S., Yu R.K. (1987). Characterization of sulfated glucuronic acid containing glycolipids reacting with IgM M-proteins in patients with neuropathy. J. Biol. Chem..

[30] Schakel K., Kannagi R., Kniep B., Goto Y., Mitsuoka C., Zwirner J., Soruri A., von Kietzell M., Rieber E. (2002). 6-Sulfo LacNAc, a novel carbohydrate modification of PSGL-1, defines an inflammatory type of human dendritic cells. Immunity.

[31] Langford R., Hurrion E., Dawson P.A. (2017). Genetics and pathophysiology of mammalian sulfate biology. J. Genet. Genomics.

[32] Gunal S., Hardman R., Kopriva S., Mueller J.W. (2019). Sulfation pathways from red to green. J. Biol. Chem..

[33] Ouyang Y., Lane W.S., Moore K.L. (1998). Tyrosylprotein sulfotransferase: Purification and molecular cloning of an enzyme that catalyzes tyrosine O-sulfation, a common posttranslational modification of eukaryotic proteins. Proc. Natl. Acad. Sci. U. S. A..

[34] Beisswanger R., Corbeil D., Vannier C., Thiele C., Dohrmann U., Kellner R., Ashman K., Niehrs C., Huttner W.B. (1998). Existence of distinct tyrosylprotein sulfotransferase genes: Molecular characterization of tyrosylprotein sulfotransferase-2. Proc. Natl. Acad. Sci. U. S. A..

[35] Kusche-Gullberg M., Kjellen L. (2003). Sulfotransferases in glycosaminoglycan biosynthesis. Curr. Opin. Struct. Biol..

[36] Seko A., Ohkura T., Ideo H., Yamashita K. (2012). Novel O-linked glycans containing 6'-sulfo-Gal/GalNAc of MUC1 secreted from human breast cancer YMB-S cells: Possible carbohydrate epitopes of KL-6(MUC1) monoclonal antibody. Glycobiology.

[37] Flowers S.A., Ali L., Lane C.S., Olin M., Karlsson N.G. (2013). Selected reaction monitoring to differentiate and relatively quantitate isomers of sulfated and unsulfated core 1 O-glycans from salivary MUC7 protein in rheumatoid arthritis. Mol. Cell. Proteomics.

[38] Narimatsu Y., Joshi H.J., Nason R., Van Coillie J., Karlsson R., Sun L., Ye Z., Chen Y.H., Schjoldager K.T., Steentoft C., Furukawa S., Bensing B.A., Sullam P.M., Thompson A.J., Paulson J.C. (2019). An atlas of human glycosylation pathways enables display of the human glycome by gene engineered cells. Mol. Cell.

[39] Narimatsu Y., Bull C., Chen Y.H., Wandall H.H., Yang Z., Clausen H. (2021). Genetic glycoengineering in mammalian cells. J. Biol. Chem..

[40] Nason R., Bull C., Konstantinidi A., Sun L., Ye Z., Halim A., Du W., Sorensen D.M., Durbesson F., Furukawa S., Mandel U., Joshi H.J., Dworkin L.A., Hansen L., David L. (2021). Display of the human mucinome with defined O-glycans by gene engineered cells. Nat. Commun..

[41] Narimatsu Y., Joshi H.J., Yang Z., Gomes C., Chen Y.H., Lorenzetti F.C., Furukawa S., Schjoldager K.T., Hansen L., Clausen H., Bennett E.P., Wandall H.H. (2018). A validated gRNA library for CRISPR/Cas9 targeting of the human glycosyltransferase genome. Glycobiology.

[42] Honke K., Tsuda M., Hirahara Y., Ishii A., Makita A., Wada Y. (1997). Molecular cloning and expression of cDNA encoding human 3'-phosphoadenylylsulfate:galactosylceramide 3'-sulfotransferase. J. Biol. Chem..

[43] Honke K., Tsuda M., Koyota S., Wada Y., Iida-Tanaka N., Ishizuka I., Nakayama J., Taniguchi N. (2001). Molecular cloning and characterization of a human beta-Gal-3'-sulfotransferase that acts on both type 1 and type 2 (Gal beta 1-3/1-4GlcNAc-R) oligosaccharides. J. Biol. Chem..

[44] Seko A., Hara-Kuge S., Yamashita K. (2001). Molecular cloning and characterization of a novel human galactose 3-O-sulfotransferase that transfers sulfate to gal beta 1-->3galNAc residue in O-glycans. J. Biol. Chem..

[45] Fukuta M., Inazawa J., Torii T., Tsuzuki K., Shimada E., Habuchi O. (1997). Molecular cloning and characterization of human keratan sulfate Gal-6-sulfotransferase. J. Biol. Chem..

[46] Fukuta M., Kobayashi Y., Uchimura K., Kimata K., Habuchi O. (1998). Molecular cloning and expression of human chondroitin 6-sulfotransferase. Biochim. Biophys. Acta.

[47] Clausen H., Stroud M., Parker J., Springer G., Hakomori S. (1988). Monoclonal antibodies directed to the blood group A associated structure, galactosyl-A: Specificity and relation to the Thomsen-Friedenreich antigen. Mol. Immunol..

[48] Nudelman E.D., Mandel U., Levery S.B., Kaizu T., Hakomori S. (1989). A series of disialogangliosides with binary 2–--3 sialosyllactosamine structure, defined by monoclonal antibody NUH2, are oncodevelopmentally regulated antigens. J. Biol. Chem..

[49] Hounsell E.F., Lawson A.M., Feeney J., Gooi H.C., Pickering N.J., Stoll M.S., Lui S.C., Feizi T. (1985). Structural analysis of the O-glycosidically linked core-region oligosaccharides of human meconium glycoproteins which express oncofoetal antigens. Eur. J. Biochem..

[50] Hassan H., Reis C.A., Bennett E.P., Mirgorodskaya E., Roepstorff P., Hollingsworth M.A., Burchell J., Taylor-Papadimitriou J., Clausen H. (2000). The lectin domain of UDP-N-acetyl-D-galactosamine: Polypeptide N-acetylgalactosaminyltransferase-T4 directs its glycopeptide specificities. J. Biol. Chem..

[51] Ideo H., Seko A., Ohkura T., Matta K.L., Yamashita K. (2002). High-affinity binding of recombinant human galectin-4 to SO(3)(-)-->3Galbeta1-->3GalNAc pyranoside. Glycobiology.

[52] Ideo H., Seko A., Yamashita K. (2005). Galectin-4 binds to sulfated glycosphingolipids and carcinoembryonic antigen in patches on the cell surface of human colon adenocarcinoma cells. J. Biol. Chem..

[53] Irungu J., Dalpathado D.S., Go E.P., Jiang H., Ha H.V., Bousfield G.R., Desaire H. (2006). Method for characterizing sulfated glycoproteins in a glycosylation site-specific fashion, using ion pairing and tandem mass spectrometry. Anal. Chem..

[54] Kuo C.W., Guu S.Y., Khoo K.H. (2018). Distinctive and complementary MS(2) fragmentation characteristics for identification of sulfated sialylated N-glycopeptides by nanoLC-MS/MS workflow. J. Am. Soc. Mass Spectrom..

[55] Kuo C.W., Khoo K.H. (2020). Strategic applications of negative-mode LC-MS/MS analyses to expedite confident mass spectrometry-based identification of multiple glycosylated peptides. Anal. Chem..

[56] Mereiter S., Balmana M., Campos D., Gomes J., Reis C.A. (2019). Glycosylation in the era of cancer-targeted therapy: Where are we heading?. Cancer Cell.

[57] Kim Y.J., Varki A. (1997). Perspectives on the significance of altered glycosylation of glycoproteins in cancer. Glycoconj. J..

[58] Steentoft C., Fuhrmann M., Battisti F., Van Coillie J., Madsen T.D., Campos D., Halim A., Vakhrushev S.Y., Joshi H.J., Schreiber H., Mandel U., Narimatsu Y. (2019). A strategy for generating cancer-specific monoclonal antibodies to aberrant O-glycoproteins: Identification of a novel dysadherin-Tn antibody. Glycobiology.

[59] Tsutsumi K., Shimakawa H., Kitagawa H., Sugahara K. (1998). Functional expression and genomic structure of human chondroitin 6-sulfotransferase. FEBS Lett..

[60] Chen Y.H., Narimatsu Y., Clausen T.M., Gomes C., Karlsson R., Steentoft C., Spliid C.B., Gustavsson T., Salanti A., Persson A., Malmstrom A., Willen D., Ellervik U., Bennett E.P., Mao Y. (2018). The GAGOme: A cell-based library of displayed glycosaminoglycans. Nat. Methods.

[61] Bull C., Joshi H.J., Clausen H., Narimatsu Y. (2020). Cell-based glycan arrays-a practical guide to dissect the human glycome. STAR Protoc..

[62] Liu J., Jin C., Cherian R.M., Karlsson N.G., Holgersson J. (2015). O-glycan repertoires on a mucin-type reporter protein expressed in CHO cell pools transiently transfected with O-glycan core enzyme cDNAs. J. Biotechnol..

[63] Gonzalez-Gil A., Porell R.N., Fernandes S.M., Wei Y., Yu H., Carroll D.J., McBride R., Paulson J.C., Tiemeyer M., Aoki K., Bochner B.S., Schnaar R.L. (2018). Sialylated keratan sulfate proteoglycans are Siglec-8 ligands in human airways. Glycobiology.

[64] Habuchi O., Matsui Y., Kotoya Y., Aoyama Y., Yasuda Y., Noda M. (1993). Purification of chondroitin 6-sulfotransferase secreted from cultured chick embryo chondrocytes. J. Biol. Chem..

[65] Torii T., Fukuta M., Habuchi O. (2000). Sulfation of sialyl N-acetyllactosamine oligosaccharides and fetuin oligosaccharides by keratan sulfate Gal-6-sulfotransferase. Glycobiology.

[66] Caval T., Tian W., Yang Z., Clausen H., Heck A.J.R. (2018). Direct quality control of glycoengineered erythropoietin variants. Nat. Commun..

[67] Caval T., de Haan N., Konstantinidi A., Vakhrushev S.Y. (2021). Quantitative characterization of O-GalNAc glycosylation. Curr. Opin. Struct. Biol..

[68] Lonowski L.A., Narimatsu Y., Riaz A., Delay C.E., Yang Z., Niola F., Duda K., Ober E.A., Clausen H., Wandall H.H., Hansen S.H., Bennett E.P., Frodin M. (2017). Genome editing using FACS enrichment of nuclease-expressing cells and indel detection by amplicon analysis. Nat. Protoc..

[69] Yang Z., Steentoft C., Hauge C., Hansen L., Thomsen A.L., Niola F., Vester-Christensen M.B., Frodin M., Clausen H., Wandall H.H., Bennett E.P. (2015). Fast and sensitive detection of indels induced by precise gene targeting. Nucleic Acids Res..

[70] Massa S.M., Cooper D.N., Leffler H., Barondes S.H. (1993). L-29, an endogenous lectin, binds to glycoconjugate ligands with positive cooperativity. Biochemistry.

[71] Steentoft C., Yang Z., Wang S., Ju T., Vester-Christensen M.B., Festari M.F., King S.L., Moremen K., Larsen I.S.B., Goth C.K., Schjoldager K.T., Hansen L., Bennett E.P., Mandel U., Narimatsu Y. (2019). A validated collection of mouse monoclonal antibodies to human glycosyltransferases functioning in mucin-type O-glycosylation. Glycobiology.

[72] Vakhrushev S.Y., Dadimov D., Peter-Katalinic J. (2009). Software platform for high-throughput glycomics. Anal. Chem..

[73] Perez-Riverol Y., Csordas A., Bai J., Bernal-Llinares M., Hewapathirana S., Kundu D.J., Inuganti A., Griss J., Mayer G., Eisenacher M., Perez E., Uszkoreit J., Pfeuffer J., Sachsenberg T., Yilmaz S. (2019). The PRIDE database and related tools and resources in 2019: Improving support for quantification data. Nucleic Acids Res..

[74] Varki A., Cummings R.D., Aebi M., Packer N.H., Seeberger P.H., Esko J.D., Stanley P., Hart G., Darvill A., Kinoshita T., Prestegard J.J., Schnaar R.L., Freeze H.H., Marth J.D., Bertozzi C.R. (2015). Symbol nomenclature for graphical representations of glycans. Glycobiology.

[75] Honke K. (2014). *Handbook of Glycosyltransferases and Related Genes*.

[76] Suzuki A., Hiraoka N., Suzuki M., Angata K., Misra A.K., McAuliffe J., Hindsgaul O., Fukuda M. (2001). Molecular cloning and expression of a novel human beta-Gal-3-O-sulfotransferase that acts preferentially on N-acetyllactosamine in N- and O-glycans. J. Biol. Chem..

[77] El-Fasakhany F.M., Uchimura K., Kannagi R., Muramatsu T. (2001). A novel human Gal-3-O-sulfotransferase: molecular cloning, characterization, and its implications in biosynthesis of (SO(4)-3)Galbeta1-4(Fucalpha1-3)GlcNAc. J. Biol. Chem..

[78] Habuchi O., Hirahara Y., Uchimura K., Fukuta M. (1996). Enzymatic sulfation of galactose residue of keratan sulfate by chondroitin 6-sulfotransferase. Glycobiology.

[79] Patnode M.L., Yu S.Y., Cheng C.W., Ho M.Y., Tegesjo L., Sakuma K., Uchimura K., Khoo K.H., Kannagi R., Rosen S.D. (2013). KSGal6ST generates galactose-6-O-sulfate in high endothelial venules but does not contribute to L-selectin-dependent lymphocyte homing. Glycobiology.

[80] Sakaguchi H., Kitagawa H., Sugahara K. (2000). Functional expression and genomic structure of human N-acetylglucosamine-6-O-sulfotransferase that transfers sulfate to beta-N-acetylglucosamine at the nonreducing end of an N-acetyllactosamine sequence. Biochim. Biophys. Acta..

[81] Mikami T., Kitagawa H. (2013). Biosynthesis and function of chondroitin sulfate. Biochim. Biophys. Acta..

[82] Fukuta M., Uchimura K., Nakashima K., Kato M., Kimata K., Shinomura T., Habuchi O. (1995). Molecular cloning and expression of chick chondrocyte chondroitin 6-sulfotransferase. J. Biol. Chem..

[83] Bistrup A., Bhakta S., Lee J.K., Belov Y.Y., Gunn M.D., Zuo F.R., Huang C.C., Kannagi R., Rosen S.D., Hemmerich S. (1999). Sulfotransferases of two specificities function in the reconstitution of high endothelial cell ligands for L-selectin. J. Cell. Biol..

[84] Lee J.K., Bistrup A., van Zante A., Rosen S.D. (2003). Activities and expression pattern of the carbohydrate sulfotransferase GlcNAc6ST-3 (I-GlcNAc6ST): functional implications. Glycobiology.

[85] Akama T.O., Misra A.K., Hindsgaul O., Fukuda M.N. (2002). Enzymatic synthesis in vitro of the disulfated disaccharide unit of corneal keratan sulfate. J. Biol. Chem..

[86] Bartes A., Bhakta S., Hemmerich S. (2001). Sulfation of endothelial mucin by corneal keratan N-acetylglucosamine 6-O-sulfotransferase (GST-4beta). Biochem. Biophys. Res. Commun..

[87] Uchimura K., Fasakhany F., Kadomatsu K., Matsukawa T., Yamakawa T., Kurosawa N., Muramatsu T. (2000). Diversity of N-acetylglucosamine-6-O-sulfotransferases: molecular cloning of a novel enzyme with different distribution and specificities. Biochem. Biophys. Res. Commun..

[88] Kitagawa H., Fujita M., Ito N., Sugahara K. (2000). Molecular cloning and expression of a novel chondroitin 6-O-sulfotransferase. J. Biol. Chem..

[89] Bhakta S., Bartes A., Bowman K.G., Kao W.M., Polsky I., Lee J.K., Cook B.N., Bruehl R.E., Rosen S.D., Bertozzi C.R., Hemmerich S. (2000). Sulfation of N-acetylglucosamine by chondroitin 6-sulfotransferase 2 (GST-5). J. Biol. Chem..

[90] Hiraoka N., Misra A., Belot F., Hindsgaul O., Fukuda M. (2001). Molecular cloning and expression of two distinct human N-acetylgalactosamine 4-O-sulfotransferases that transfer sulfate to GalNAc beta 1-->4GlcNAc beta 1-->R in both N- and O-glycans. Glycobiology.

[91] Xia G., Evers M.R., Kang H.G., Schachner M., Baenziger J.U. (2000). Molecular cloning and expression of the pituitary glycoprotein hormone N-acetylgalactosamine-4-O-sulfotransferase. J. Biol. Chem..

[92] Ong E., Yeh J.C., Ding Y., Hindsgaul O., Fukuda M. (1998). Expression cloning of a human sulfotransferase that directs the synthesis of the HNK-1 glycan on the neural cell adhesion molecule and glycolipids. J. Biol. Chem..

[93] Hiraoka N., Nakagawa H., Ong E., Akama T.O., Fukuda M.N., Fukuda M. (2000). Molecular cloning and expression of two distinct human chondroitin 4-O-sulfotransferases that belong to the HNK-1 sulfotransferase gene family. J. Biol. Chem..

[94] Okuda T., Mita S., Yamauchi S., Matsubara T., Yagi F., Yamamori D., Fukuta M., Kuroiwa A., Matsuda Y., Habuchi O. (2000). Molecular cloning, expression, and chromosomal mapping of human chondroitin 4-sulfotransferase, whose expression pattern in human tissues is different from that of chondroitin 6-sulfotransferase. J. Biochem..

[95] Kang H.G., Evers M.R., Xia G., Baenziger J.U., Schachner M. (2002). Molecular cloning and characterization of chondroitin-4-O-sulfotransferase-3. A novel member of the HNK-1 family of sulfotransferases. J. Biol. Chem..

[96] Pacheco B., Maccarana M., Malmstrom A. (2009). Dermatan 4-O-sulfotransferase 1 is pivotal in the formation of iduronic acid blocks in dermatan sulfate. Glycobiology.

[97] Ohtake S., Ito Y., Fukuta M., Habuchi O. (2001). Human N-acetylgalactosamine 4-sulfate 6-O-sulfotransferase cDNA is related to human B cell recombination activating gene-associated gene. J. Biol. Chem..

[98] Ohtake S., Kimata K., Habuchi O. (2003). A unique nonreducing terminal modification of chondroitin sulfate by N-acetylgalactosamine 4-sulfate 6-o-sulfotransferase. J. Biol. Chem..

[99] Esko J.D., Selleck S.B. (2002). Order out of chaos: assembly of ligand binding sites in heparan sulfate. Annu. Rev. Biochem..

[100] Shukla D., Liu J., Blaiklock P., Shworak N.W., Bai X., Esko J.D., Cohen G.H., Eisenberg R.J., Rosenberg R.D., Spear P.G. (1999). A novel role for 3-O-sulfated heparan sulfate in herpes simplex virus 1 entry. Cell.

[101] Xia G., Chen J., Tiwari V., Ju W., Li J.P., Malmstrom A., Shukla D., Liu J. (2002). Heparan sulfate 3-O-sulfotransferase isoform 5 generates both an antithrombin-binding site and an entry receptor for herpes simplex virus, type 1. J. Biol. Chem..

[102] Xu D., Tiwari V, Xia G., Clement C., Shukla D., Liu J. (2005). Characterization of heparan sulphate 3-O-sulphotransferase isoform 6 and its role in assisting the entry of herpes simplex virus type 1. Biochem. J..

[103] Pikas D.S., Eriksson I., Kjellen L. (2000). Overexpression of different isoforms of glucosaminyl N-deacetylase/N-sulfotransferase results in distinct heparan sulfate N-sulfation patterns. Biochemistry.

[104] van den Born J., Pikas D.S., Pisa B.J., Eriksson I., Kjellen L., Berden J.H. (2003). Antibody-based assay for N-deacetylase activity of heparan sulfate/heparin N-deacetylase/N-sulfotransferase (NDST): novel characteristics of NDST-1 and -2. Glycobiology.

[105] Baietti M.F., Zhang Z., Mortier E., Melchior A., Degeest G., Geeraerts A., Ivarsson Y., Depoortere F., Coomans C., Vermeiren E., Zimmermann P., David G. (2012). Syndecan-syntenin-ALIX regulates the biogenesis of exosomes. Nat. Cell. Biol..

[106] Duncan M.B., Liu M., Fox C., Liu J. (2006). Characterization of the N-deacetylase domain from the heparan sulfate N-deacetylase/N-sulfotransferase 2. Biochem. Biophys. Res. Commun..

[107] Aikawa J., Esko J.D. (1999). Molecular cloning and expression of a third member of the heparan sulfate/heparin GlcNAc N-deacetylase/ N-sulfotransferase family. J. Biol. Chem..

[108] Aikawa J., Grobe K., Tsujimoto M., Esko J.D. (2001). Multiple isozymes of heparan sulfate/heparin GlcNAc N-deacetylase/GlcN N-sulfotransferase. Structure and activity of the fourth member, NDST4. J. Biol. Chem..

[109] Tornberg J., Sykiotis G.P., Keefe K., Plummer L., Hoang X., Hall J.E., Quinton R., Seminara S.B., Hughes V., Van Vliet G., Van Uum S., Crowley W.F., Habuchi H., Kimata K., Pitteloud N., Bulow H.E. (2011). Heparan sulfate 6-O-sulfotransferase 1, a gene involved in extracellular sugar modifications, is mutated in patients with idiopathic hypogonadotrophic hypogonadism. Proc. Natl. Acad. Sci. U. S. A..

[110] Habuchi H., Miyake G., Nogami K., Kuroiwa A., Matsuda Y., Kusche-Gullberg M., Habuchi O., Tanaka M., Kimata K. (2003). Biosynthesis of heparan sulphate with diverse structures and functions: two alternatively spliced forms of human heparan sulphate 6-O-sulphotransferase-2 having different expression patterns and properties. Biochem. J..

[111] Paganini L., Hadi L.A., Chetta M., Rovina D., Fontana L., Colapietro P., Bonaparte E., Pezzani L., Marchisio P., Tabano S.M., Costanza J., Sirchia S.M., Riboni L., Milani D., Miozzo M. (2019). A HS6ST2 gene variant associated with X-linked intellectual disability and severe myopia in two male twins. Clin. Genet..

[112] Sugahara K., Kitagawa H. (2000). Recent advances in the study of the biosynthesis and functions of sulfated glycosaminoglycans. Curr. Opin. Struct. Biol..

