# Supplementary material for: Installation of O-glycan sulfation capacities in human HEK293 cells for display of sulfated mucins
Source: J Biol Chem. 2021 Dec 24;298(2):101382. doi: 10.1016/j.jbc.2021.101382 (PMC8789585; doi:10.1016/j.jbc.2021.101382)
Supplement: Supplemental Figures S1–S8 and Tables S1, S2 [file mmc1.docx]

**SUPPORTING INFORMATION**

**Installation of O-glycan sulfation capacities in human HEK293 cells**

Lingbo Sun^1,2^, Andriana Konstantinidi^1^, Zilu Ye^1^, Rebecca Nason^1^, Yuecheng Zhang^3^, Christian Büll^1^, Barbro Kahl-Knutson^4^, Lars Hansen^1^, Hakon Leffler^4^, Sergey Y. Vakhrushev^1^, Zhang Yang^1^, Henrik Clausen^1,^*, and Yoshiki Narimatsu^1,^*.

**List of Supporting Figures, Tables and File**

**Figure S1. Rainbow depiction of sulfotransferases involved in sulfation of GAGs and non-GAG glycans.**

**Figure S2. Graphic depiction of the genetic engineering strategy for 3-O-sulfo-T and 6-O-sulfo-Tn O-glycosylation.**

**Figure S3.** **Figure S3.** **Summary of RNAseq analysis of sulfotransferase gene expression in HEK293 6E WT cells.**

**Figure S4. Analysis of CHST1 and CHST3 KI engineered HEK293 cells with lectins and mAbs.**

**Figure S5. Graphic depiction of the secreted mucin TR reporter constructs for MUC1, MUC2 and MUC7.**

**Figure S6. SDS-PAGE Coomassie staining analysis of the purified MUC1 reporter engineered with KI of GAL3ST2 and GAL3ST4 for 3-O-sulfo-T O-glycosylation.**

**Figure S7. SDS-PAGE Coomassie staining analysis of MUC2 and MUC7 TR reporters produced in glycoengineered HEK293 cells with KI of GAL3ST2 and GAL3ST4.**

**Figure S8. Bottom up site specific analysis of MUC1 TR glycopeptides after Endo-AspN digestion.**

**Table S1. Human sulfotransferases involved in sulfation of GAGs and non-GAG glycans.**

**Table S2. List of all engineered HEK293 cells used in this study.**

**Supporting File 1. Peak assignment of intact mass spectra related to Figure 4.**

**
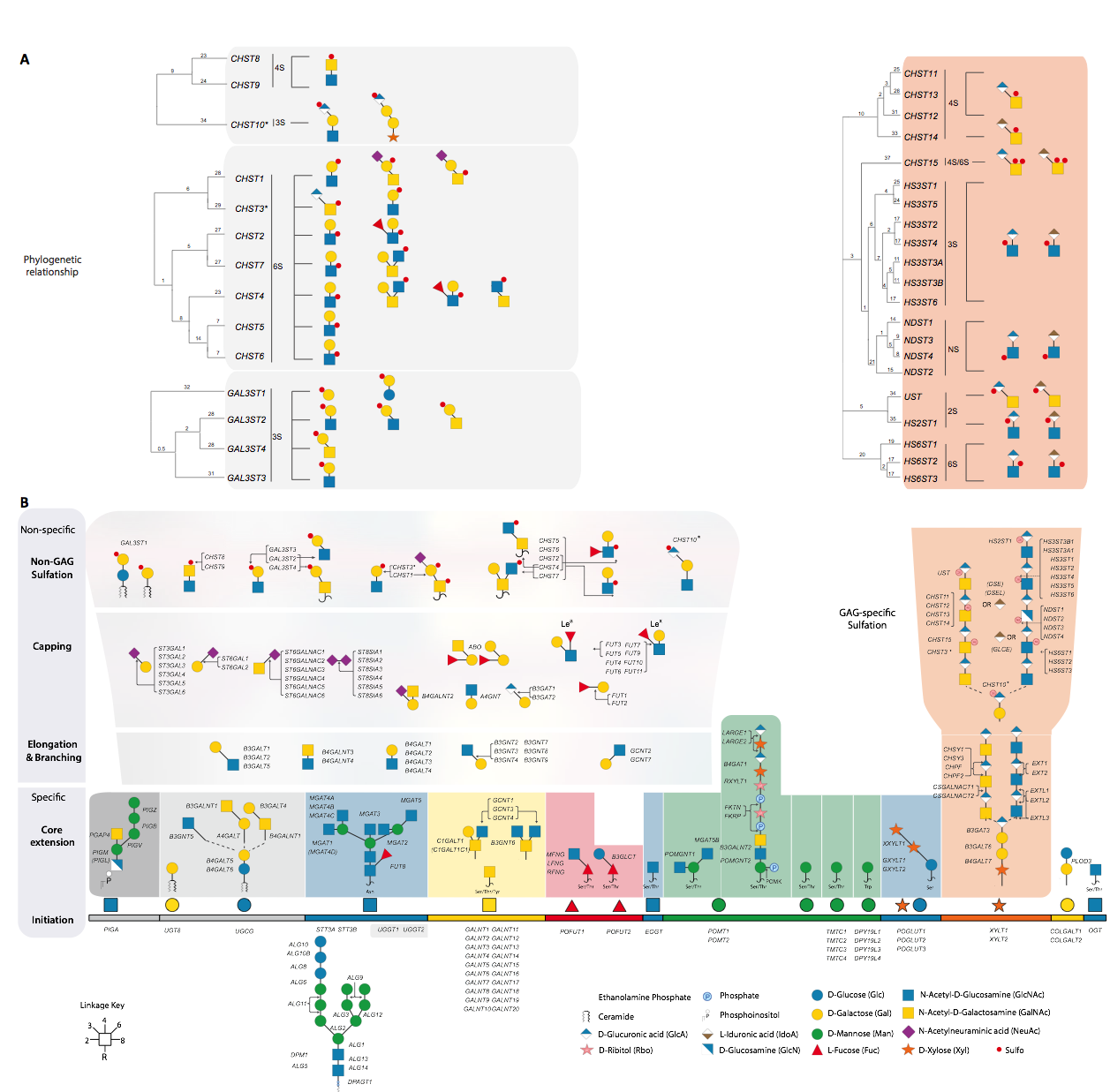
**

**Figure S1. Rainbow depiction of sulfotransferases involved in sulfation of GAGs and non-GAG glycans.** *A*, Sulfotransferase genes are arranged by phylogenetic analysis and separated in the pathway-specific related GAG biosynthesis (22 genes) and pathway-nonspecific steps (13 genes) with function based on recent publication (Please see references in Table S1). *Sulfotransferases that appear twice in the figure due to dual pathway-specificity. *B*, Rainbow depiction of the 16 distinct human glycosylation pathways with the major structural elements of the glycans and the assigned (predicted) biosynthetic roles for glycosyltransferases as well as carbohydrate sulfotransferases as we showed previously (1,2). Glycan symbols are drawn according to the SNFG format (3).


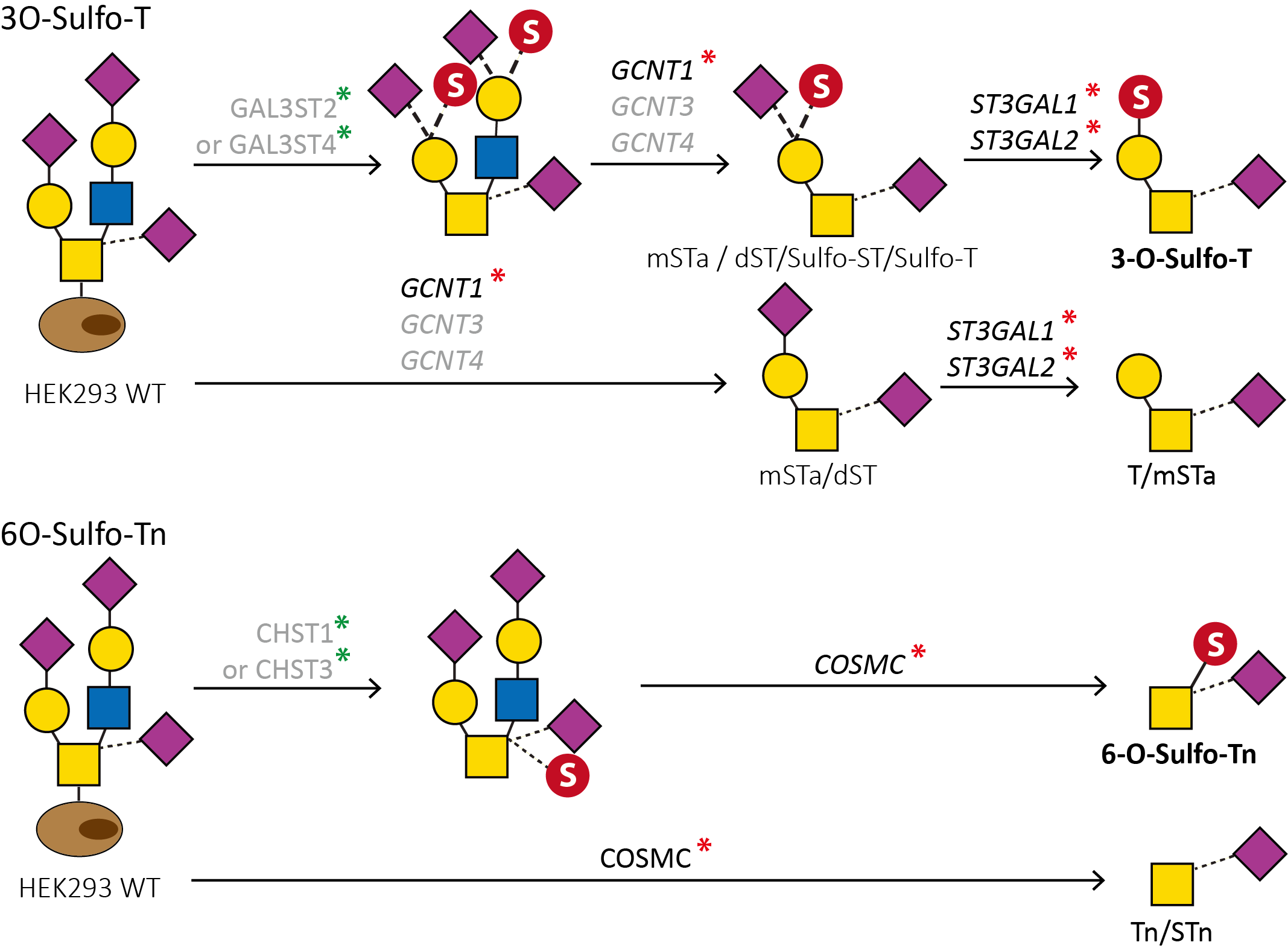


**Figure S2. Graphic depiction of the genetic engineering strategy for 3-O-sulfo-T and 6-O-sulfo-Tn O-glycosylation.** Genes in black letters are endogenously expressed in HEK293 6E WT cells, while grey letter indicate genes not expressed as per RNAseq transcriptome analysis (1) (**Fig. S3**). KI of sulfotransferase complementary DNA (capitalized with green asterisk) and following KO (capitalized and italicized with red asterisk) of glycosyltransferase genes controlling core2 (*GCNT1*), α2-3 sialylation of core1 (*ST3GAL1/2*), and core1 O-glycosylation (*C1GALT1*/*COSMC*) are shown. The engineering strategy was designed to first generate stable KI of sulfotransferases and then perform KO of key glycosyltransferase genes to eliminate potential competition with sulfation.


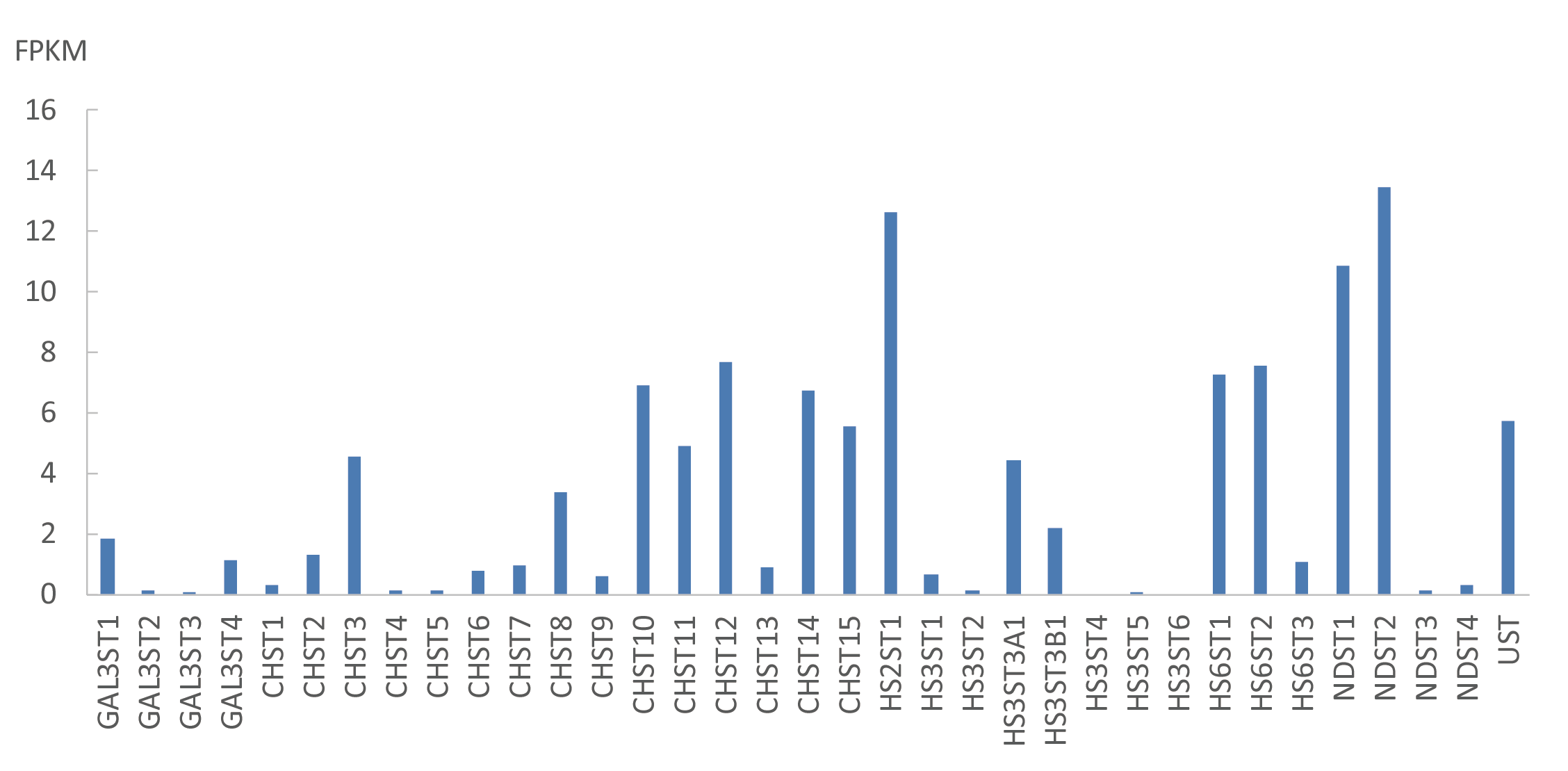


**Figure S3.** **Summary of RNAseq analysis of sulfotransferase gene expression in HEK293 6E WT cells.** RNAseq analysis was performed with HEK293 6E cells as previously reported (1). Genes known to the Golgi sulfotransferase are listed.


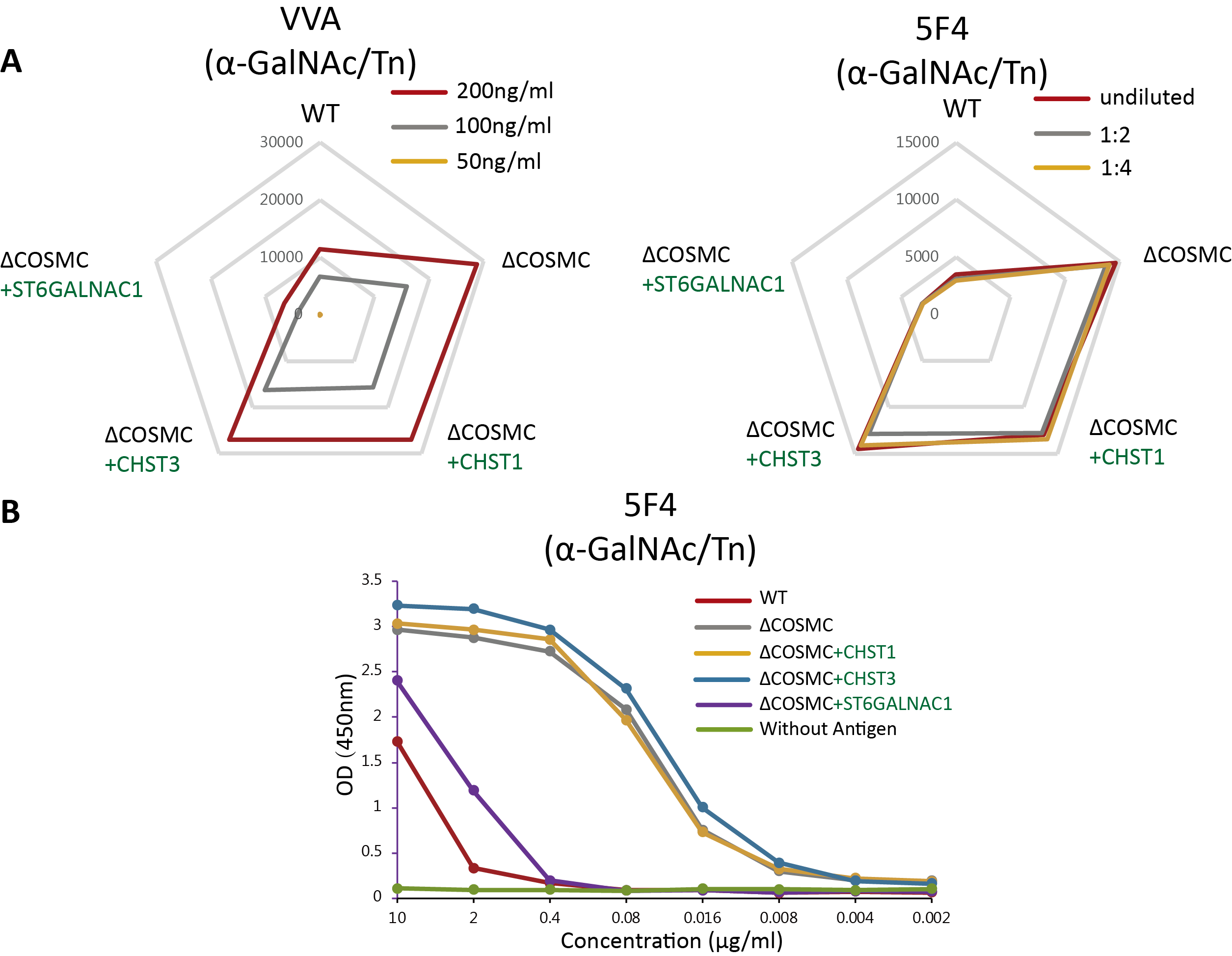


**Figure S4. Analysis of CHST1 and CHST3 KI engineered HEK293 cells with lectins and mAbs.** *A*, Flow cytometry analysis of HEK293 cells stably engineered with KO (∆) and KI (+) as indicated were probed with VVA lectin and the anti-Tn mAb 5F4 at different concentrations (color coded). Radar charts show mean fluorescence intensities (MFIs) and solid/dashed lines represent binding with and without neuraminidase treatment. *B*, ELISA analysis on purified glycoengineered MUC1 reporters as indicated with 5F4 mAb. Both FACS and ELISA results represent single experiments and independent experiments were performed at least three times with similar results.


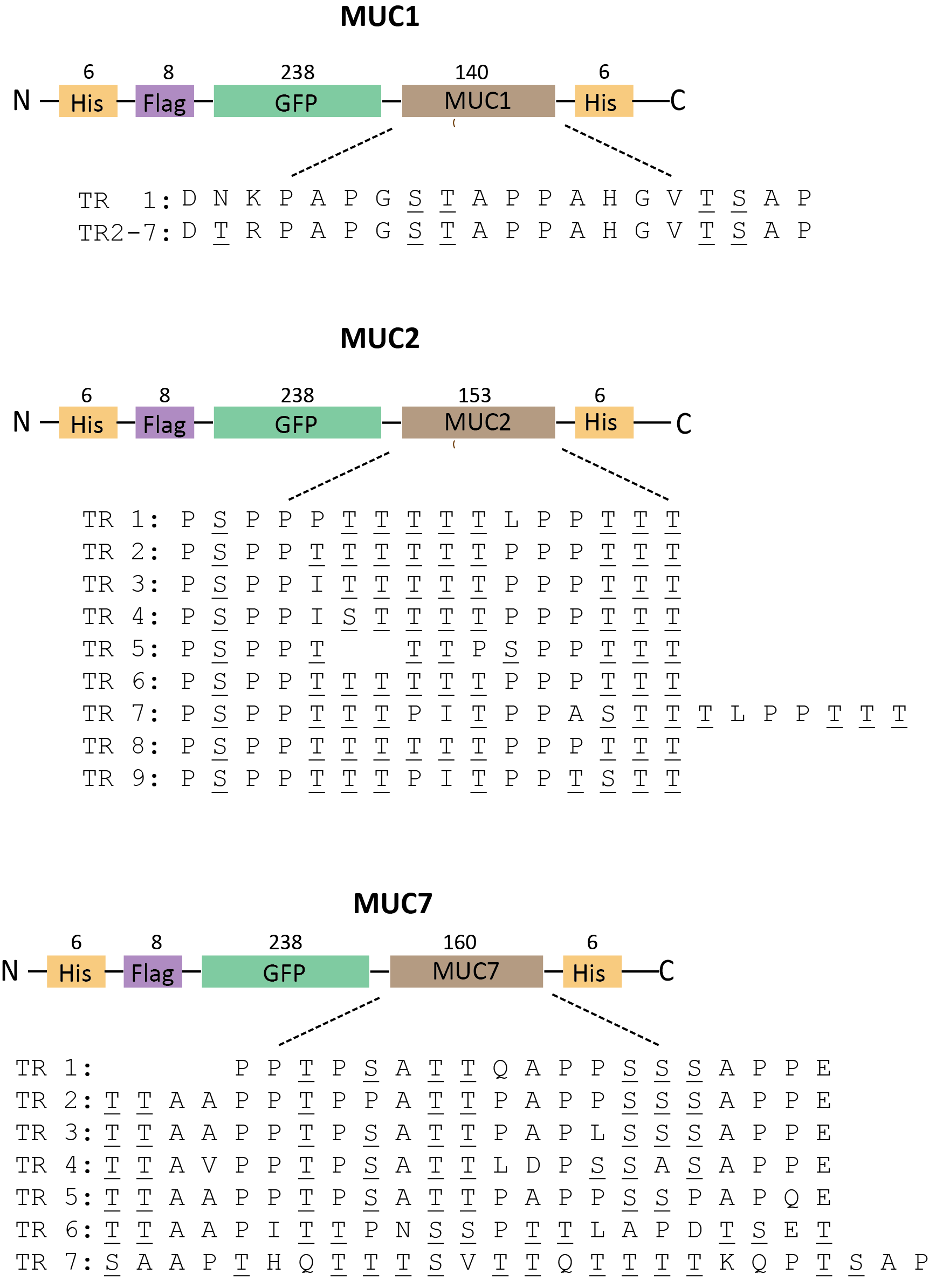


**Figure S5. Graphic depiction of the secreted mucin TR reporter constructs for MUC1, MUC2 and MUC7.** Illustration of the secreted mucin reporter design contains an N-terminal 6xHis and FLAG-tag and EGFP followed by different mucin TR domains of MUC1, MUC2 and MUC7 and a second C-terminal 6xHis tag. The number of amino acids for each domain is shown in above. Full sequences of the TRs are shown with the repeated number of TR.


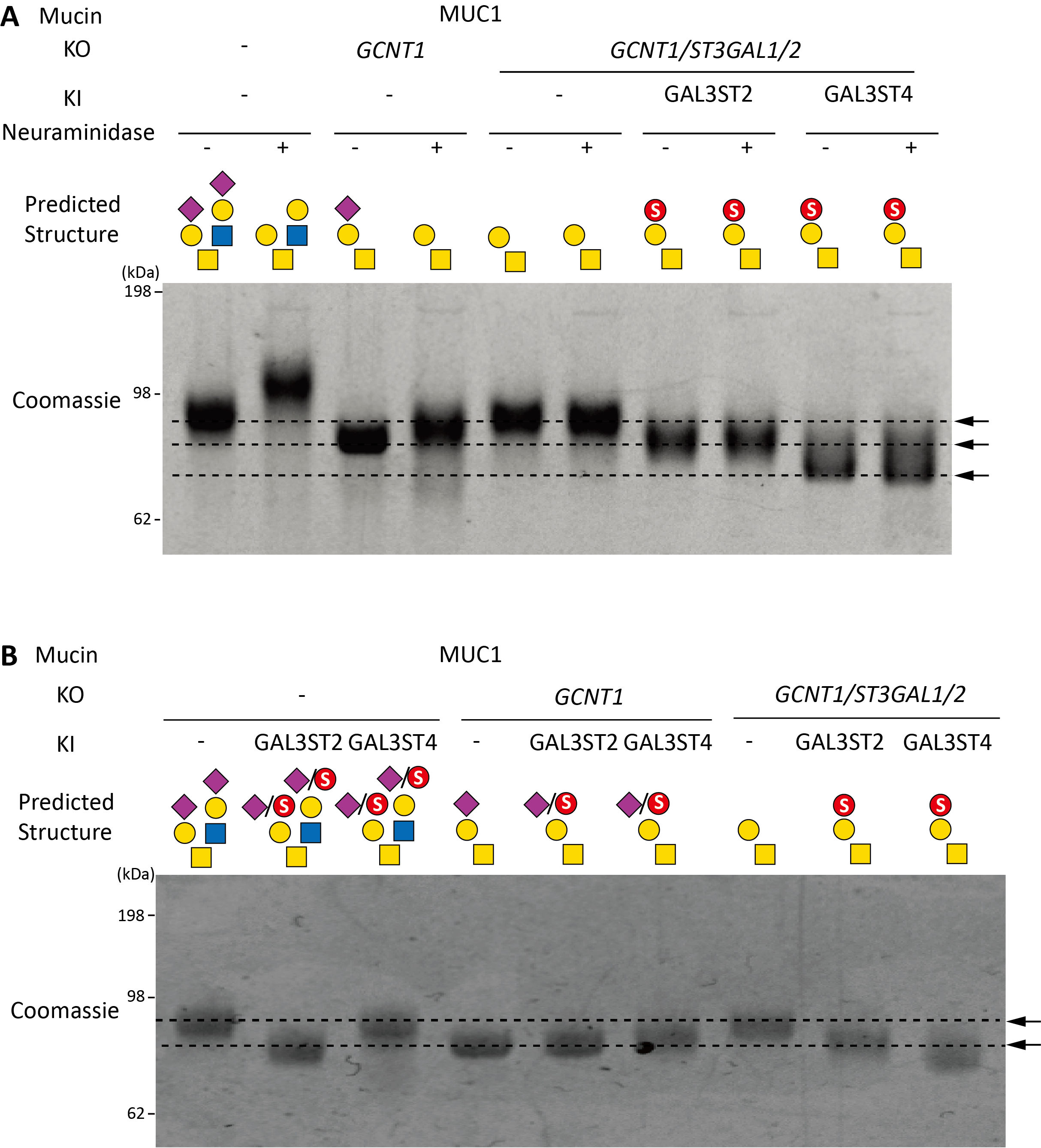


**Figure S6. SDS-PAGE Coomassie staining analysis of the purified MUC1 reporter engineered with KI of GAL3ST2 and GAL3ST4 for 3-O-sulfo-T O-glycosylation.** *A,* SDS-PAGE Coomassie of the purified MUC1 reporter with and without neuraminidase treatment as indicated. The predicted O-glycan structures produced by the engineering are shown. *B,* SDS-PAGE of the purified MUC1 reporter with and without sulfotransferase (GAL3ST2 or GAL3ST4) in combination with KO of *GCNT1* and *ST3GAL1/2*. Independent experiments were performed at least three times with similar results.


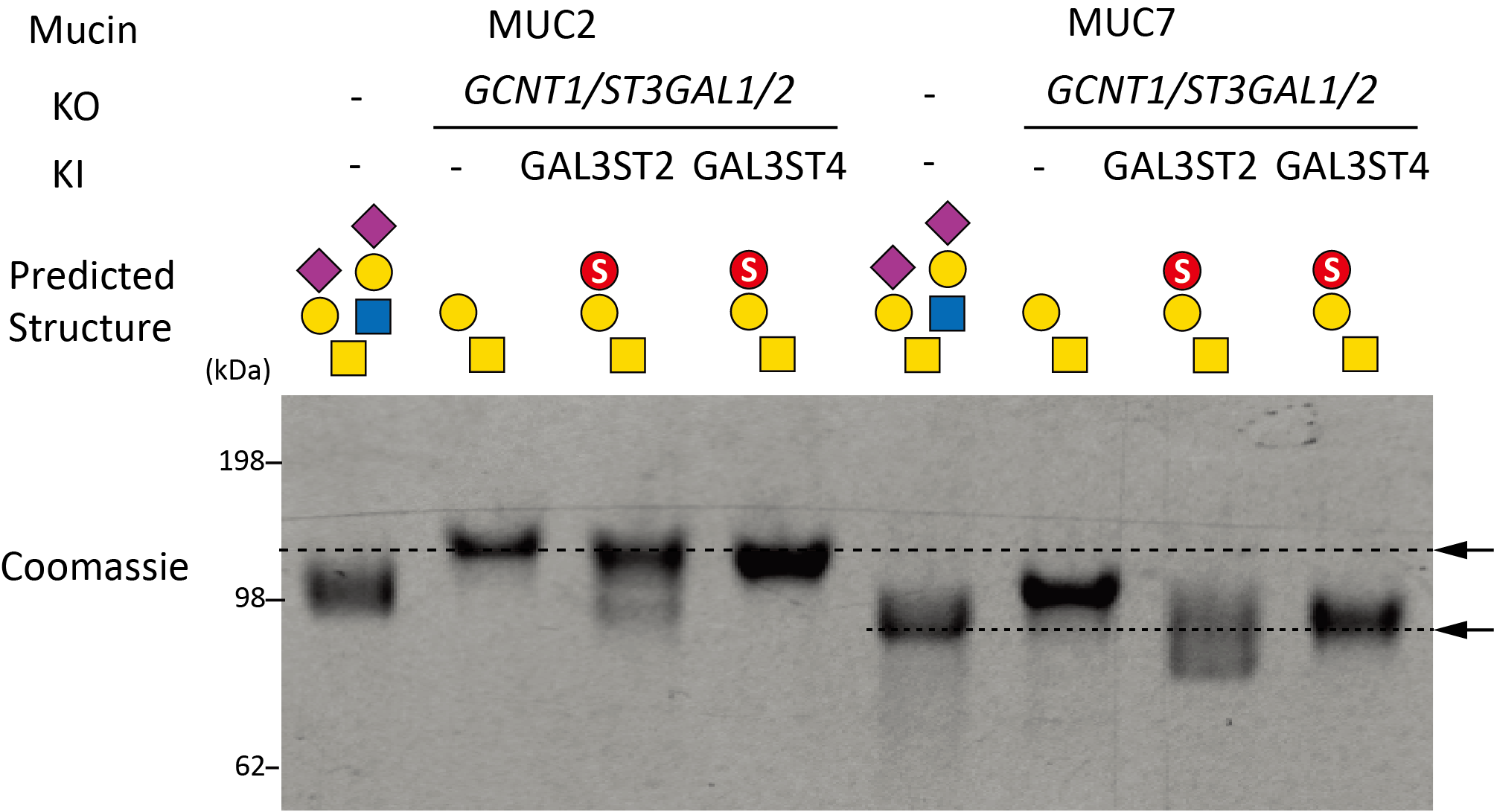


**Figure S7. SDS-PAGE Coomassie staining analysis of MUC2 and MUC7 TR reporters produced in glycoengineered HEK293 cells with KI of GAL3ST2 and GAL3ST4.** Independent experiments were performed at least three times with similar results.


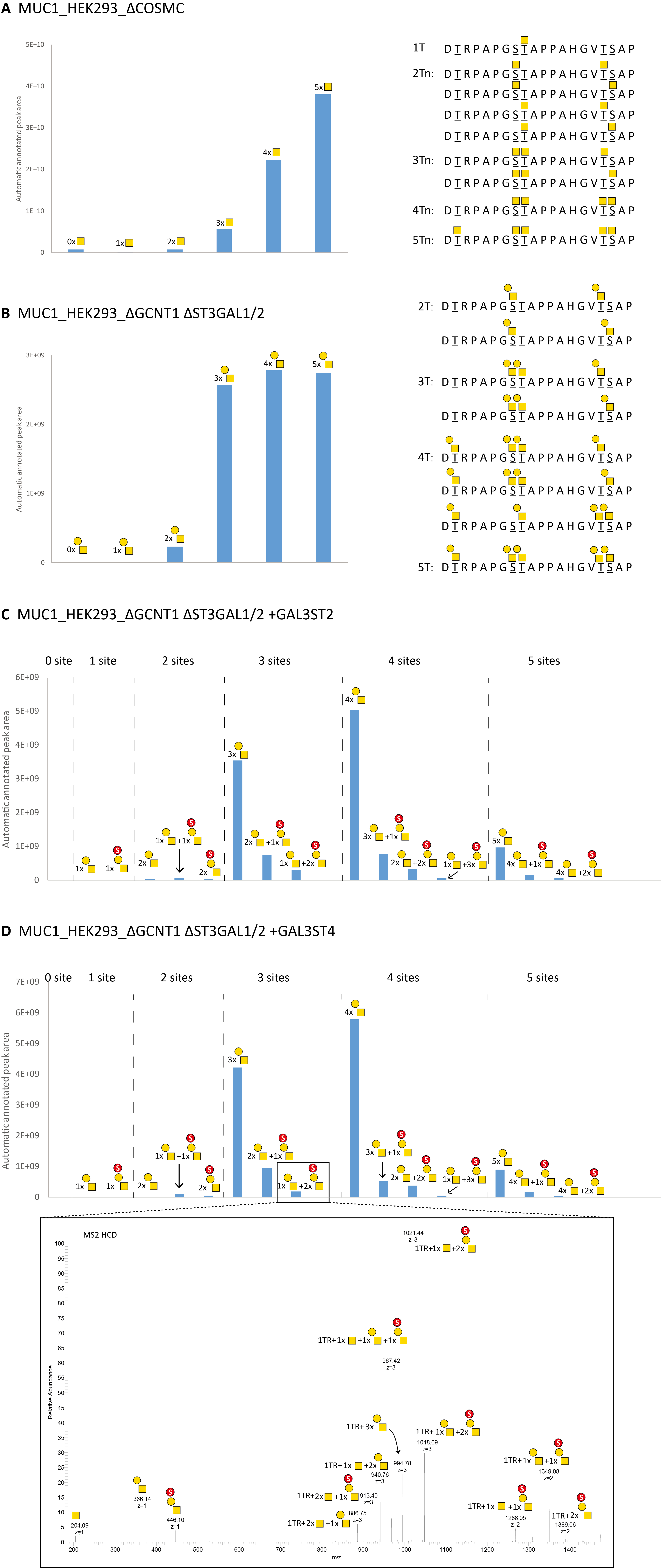


**Figure S8. Bottom up site specific analysis of MUC1 TR glycopeptides after Endo-AspN digestion.** The MUC1-TR glycopeptide precursors were subjected to ETD or HCD MS/MS analysis to determine glycosylation site localization. The sites of Tn (GalNAc) and T (Gal-GalNAc) are depicted with the MUC1-TRs. The bar graphs are based on the automatic annotated peak areas from extracted-ion chromatograms (XIC) of MUC1 TRs AspN-digested peptide (DTRPAPGSTAPPAHGVTSAP) expressed in HEK293^KO^ *^COSMC^*(A), HEK293^KO^ *^GCNT1, ST3GAL1/2^*(B), HEK293^KO^ *^GCNT1 ST3GAL1/2^*^; KI GAL3ST2^(C) and HEK293^KO^ *^GCNT1, ST3GAL1/2^*^; KI GAL3ST4^(D). Zoom in spectra in. *D,* shows the HCD MS/MS analysis of 1 core1 + 2 sulfated core1. Independent experiments were performed at least three times with similar results.

**Table S1. Human sulfotransferases involved in sulfation of GAGs and non-GAG glycans.**

| **Sulfotransferases** | **Alternative Names** | **Ref.** |
| --- | --- | --- |
| GAL3ST1 | CST | (4,5) |
| GAL3ST2 | CP3ST | (5,6) |
| GAL3ST3 | / | (5,7,8) |
| GAL3ST4 | / | (5,9) |
| CHST1 | KS6ST, KSGal6ST, KSST | (10-13) |
| CHST2 | GlcNAc6ST-1, Gn6ST-1 | (14) |
| CHST3 | C6ST | (15-19) |
| CHST4 | GlcNAc6ST-2, Gn6ST-2, HEC-GlcNAc6ST, LSST | (20) |
| CHST5 | GlcNAc6ST-3, Gn6ST-3, I-GlcNAc6ST, hIGn6ST | (21,22). |
| CHST6 | GlcNAc6ST-5, Gn6ST-5, hCGn6ST | (22,23) |
| CHST7 | GlcNAc6ST-4, Gn6ST-4, C6ST-2 | (24-26) |
| CHST8 | GalNAc4ST-1 | (27,28) |
| CHST9 | GalNAc4ST-2 | (27) |
| CHST10 | HNK1ST | (29) |
| CHST11 | C4ST | (19,30,31) |
| CHST12 | C4ST-2 | (19,30) |
| CHST13 | C4ST-3 | (19,32) |
| CHST14 | D4ST-1, hD4ST1 | (19,33) |
| CHST15 | hBRAG, GalNAc4S-6ST | (16,33-35) (19) |
| HS3ST1 | 3-OST-1, h3-OST-1 | (19,35,36) |
| HS3ST2 | 3-OST-2, h3-OST-2 | (19,36) |
| HS3ST3A | 3-OST-3, h3-OST-3 | (19,36,37) |
| HS3ST3B | 3-OST-3B, h3-OST-3B | (19,37) |
| HS3ST4 | 3-OST-4, h3-OST-4 | (19,36) |
| HS3ST5 | 3-OST-5, h3-OST-5 | (19,36,38) |
| HS3ST6 | 3-OST-6, h3-OST-6 | (19,36,39) |
| NDST1 | HSST1 | (19,36,40-42) |
| NDST2 | HSST2 | (19,36,40-43) |
| NDST3 | HSST3 | (19,36,44) |
| NDST4 | HSST4 | (19,36,45) |
| HS2ST1 | 2OST, HS2ST | (19,36) |
| HS6ST1 | / | (19,36,46,47) |
| HS6ST2 | / | (19,36,48,49) |
| HS6ST3 | / | (19,36) |
| UST | DS2ST | (16,50) |

**Table S2 List of all engineered HEK293 cells used in this study.**

**References**

1. Narimatsu, Y., Joshi, H. J., Nason, R., Van Coillie, J., Karlsson, R., Sun, L., Ye, Z., Chen, Y. H., Schjoldager, K. T., Steentoft, C., Furukawa, S., Bensing, B. A., Sullam, P. M., Thompson, A. J., Paulson, J. C., Bull, C., Adema, G. J., Mandel, U., Hansen, L., Bennett, E. P., Varki, A., Vakhrushev, S. Y., Yang, Z., and Clausen, H. (2019) An Atlas of Human Glycosylation Pathways Enables Display of the Human Glycome by Gene Engineered Cells. *Mol. Cell.* **75**, 394-407

2. Schjoldager, K. T., Narimatsu, Y., Joshi, H. J., and Clausen, H. (2020) Global view of human protein glycosylation pathways and functions. *Nat. Rev. Mol. Cell. Biol.* **21**, 729-749

3. Varki, A., Cummings, R. D., Aebi, M., Packer, N. H., Seeberger, P. H., Esko, J. D., Stanley, P., Hart, G., Darvill, A., Kinoshita, T., Prestegard, J. J., Schnaar, R. L., Freeze, H. H., Marth, J. D., Bertozzi, C. R., Etzler, M. E., Frank, M., Vliegenthart, J. F., Lutteke, T., Perez, S., Bolton, E., Rudd, P., Paulson, J., Kanehisa, M., Toukach, P., Aoki-Kinoshita, K. F., Dell, A., Narimatsu, H., York, W., Taniguchi, N., and Kornfeld, S. (2015) Symbol Nomenclature for Graphical Representations of Glycans. *Glycobiology.* **25**, 1323-1324

4. Honke, K., Tsuda, M., Hirahara, Y., Ishii, A., Makita, A., and Wada, Y. (1997) Molecular cloning and expression of cDNA encoding human 3'-phosphoadenylylsulfate:galactosylceramide 3'-sulfotransferase. *J. Biol. Chem.* **272**, 4864-4868

5. Honke, K. (2014) Galactose-3-O-Sulfotransferase 1-4 (GAL3ST1-4). in *Handbook of Glycosyltransferases and Related Genes*. pp 1123-1132

6. Honke, K., Tsuda, M., Koyota, S., Wada, Y., Iida-Tanaka, N., Ishizuka, I., Nakayama, J., and Taniguchi, N. (2001) Molecular cloning and characterization of a human beta-Gal-3'-sulfotransferase that acts on both type 1 and type 2 (Gal beta 1-3/1-4GlcNAc-R) oligosaccharides. *J. Biol. Chem.* **276**, 267-274

7. Suzuki, A., Hiraoka, N., Suzuki, M., Angata, K., Misra, A. K., McAuliffe, J., Hindsgaul, O., and Fukuda, M. (2001) Molecular cloning and expression of a novel human beta-Gal-3-O-sulfotransferase that acts preferentially on N-acetyllactosamine in N- and O-glycans. *J. Biol. Chem.* **276**, 24388-24395

8. El-Fasakhany, F. M., Uchimura, K., Kannagi, R., and Muramatsu, T. (2001) A novel human Gal-3-O-sulfotransferase: molecular cloning, characterization, and its implications in biosynthesis of (SO(4)-3)Galbeta1-4(Fucalpha1-3)GlcNAc. *J. Biol. Chem.* **276**, 26988-26994

9. Seko, A., Hara-Kuge, S., and Yamashita, K. (2001) Molecular cloning and characterization of a novel human galactose 3-O-sulfotransferase that transfers sulfate to gal beta 1-->3galNAc residue in O-glycans. *J. Biol. Chem.* **276**, 25697-25704

10. Fukuta, M., Inazawa, J., Torii, T., Tsuzuki, K., Shimada, E., and Habuchi, O. (1997) Molecular cloning and characterization of human keratan sulfate Gal-6-sulfotransferase. *J. Biol. Chem.* **272**, 32321-32328

11. Seko, A., Ohkura, T., Ideo, H., and Yamashita, K. (2012) Novel O-linked glycans containing 6'-sulfo-Gal/GalNAc of MUC1 secreted from human breast cancer YMB-S cells: possible carbohydrate epitopes of KL-6(MUC1) monoclonal antibody. *Glycobiology.* **22**, 181-195

12. Habuchi, O., Hirahara, Y., Uchimura, K., and Fukuta, M. (1996) Enzymatic sulfation of galactose residue of keratan sulfate by chondroitin 6-sulfotransferase. *Glycobiology.* **6**, 51-57

13. Patnode, M. L., Yu, S. Y., Cheng, C. W., Ho, M. Y., Tegesjo, L., Sakuma, K., Uchimura, K., Khoo, K. H., Kannagi, R., and Rosen, S. D. (2013) KSGal6ST generates galactose-6-O-sulfate in high endothelial venules but does not contribute to L-selectin-dependent lymphocyte homing. *Glycobiology.* **23**, 381-394

14. Sakaguchi, H., Kitagawa, H., and Sugahara, K. (2000) Functional expression and genomic structure of human N-acetylglucosamine-6-O-sulfotransferase that transfers sulfate to beta-N-acetylglucosamine at the nonreducing end of an N-acetyllactosamine sequence. *Biochim. Biophys. Acta.* **1523**, 269-276

15. Fukuta, M., Kobayashi, Y., Uchimura, K., Kimata, K., and Habuchi, O. (1998) Molecular cloning and expression of human chondroitin 6-sulfotransferase. *Biochim. Biophys. Acta.* **1399**, 57-61

16. Mikami, T., and Kitagawa, H. (2013) Biosynthesis and function of chondroitin sulfate. *Biochim. Biophys. Acta.* **1830**, 4719-4733

17. Habuchi, O., Suzuki, Y., and Fukuta, M. (1997) Sulfation of sialyl lactosamine oligosaccharides by chondroitin 6-sulfotransferase. *Glycobiology.* **7**, 405-412

18. Fukuta, M., Uchimura, K., Nakashima, K., Kato, M., Kimata, K., Shinomura, T., and Habuchi, O. (1995) Molecular cloning and expression of chick chondrocyte chondroitin 6-sulfotransferase. *J. Biol. Chem.* **270**, 18575-18580

19. Chen, Y. H., Narimatsu, Y., Clausen, T. M., Gomes, C., Karlsson, R., Steentoft, C., Spliid, C. B., Gustavsson, T., Salanti, A., Persson, A., Malmstrom, A., Willen, D., Ellervik, U., Bennett, E. P., Mao, Y., Clausen, H., and Yang, Z. (2018) The GAGOme: a cell-based library of displayed glycosaminoglycans. *Nat. Methods.* **15**, 881-888

20. Bistrup, A., Bhakta, S., Lee, J. K., Belov, Y. Y., Gunn, M. D., Zuo, F. R., Huang, C. C., Kannagi, R., Rosen, S. D., and Hemmerich, S. (1999) Sulfotransferases of two specificities function in the reconstitution of high endothelial cell ligands for L-selectin. *J. Cell. Biol.* **145**, 899-910

21. Lee, J. K., Bistrup, A., van Zante, A., and Rosen, S. D. (2003) Activities and expression pattern of the carbohydrate sulfotransferase GlcNAc6ST-3 (I-GlcNAc6ST): functional implications. *Glycobiology.* **13**, 245-254

22. Akama, T. O., Misra, A. K., Hindsgaul, O., and Fukuda, M. N. (2002) Enzymatic synthesis in vitro of the disulfated disaccharide unit of corneal keratan sulfate. *J. Biol. Chem.* **277**, 42505-42513

23. Bartes, A., Bhakta, S., and Hemmerich, S. (2001) Sulfation of endothelial mucin by corneal keratan N-acetylglucosamine 6-O-sulfotransferase (GST-4beta). *Biochem. Biophys. Res. Commun.* **282**, 928-933

24. Uchimura, K., Fasakhany, F., Kadomatsu, K., Matsukawa, T., Yamakawa, T., Kurosawa, N., and Muramatsu, T. (2000) Diversity of N-acetylglucosamine-6-O-sulfotransferases: molecular cloning of a novel enzyme with different distribution and specificities. *Biochem. Biophys. Res. Commun.* **274**, 291-296

25. Kitagawa, H., Fujita, M., Ito, N., and Sugahara, K. (2000) Molecular cloning and expression of a novel chondroitin 6-O-sulfotransferase. *J. Biol. Chem.* **275**, 21075-21080

26. Bhakta, S., Bartes, A., Bowman, K. G., Kao, W. M., Polsky, I., Lee, J. K., Cook, B. N., Bruehl, R. E., Rosen, S. D., Bertozzi, C. R., and Hemmerich, S. (2000) Sulfation of N-acetylglucosamine by chondroitin 6-sulfotransferase 2 (GST-5). *J. Biol. Chem.* **275**, 40226-40234

27. Hiraoka, N., Misra, A., Belot, F., Hindsgaul, O., and Fukuda, M. (2001) Molecular cloning and expression of two distinct human N-acetylgalactosamine 4-O-sulfotransferases that transfer sulfate to GalNAc beta 1-->4GlcNAc beta 1-->R in both N- and O-glycans. *Glycobiology.* **11**, 495-504

28. Xia, G., Evers, M. R., Kang, H. G., Schachner, M., and Baenziger, J. U. (2000) Molecular cloning and expression of the pituitary glycoprotein hormone N-acetylgalactosamine-4-O-sulfotransferase. *J. Biol. Chem.* **275**, 38402-38409

29. Ong, E., Yeh, J. C., Ding, Y., Hindsgaul, O., and Fukuda, M. (1998) Expression cloning of a human sulfotransferase that directs the synthesis of the HNK-1 glycan on the neural cell adhesion molecule and glycolipids. *J. Biol. Chem.* **273**, 5190-5195

30. Hiraoka, N., Nakagawa, H., Ong, E., Akama, T. O., Fukuda, M. N., and Fukuda, M. (2000) Molecular cloning and expression of two distinct human chondroitin 4-O-sulfotransferases that belong to the HNK-1 sulfotransferase gene family. *J. Biol. Chem.* **275**, 20188-20196

31. Okuda, T., Mita, S., Yamauchi, S., Matsubara, T., Yagi, F., Yamamori, D., Fukuta, M., Kuroiwa, A., Matsuda, Y., and Habuchi, O. (2000) Molecular cloning, expression, and chromosomal mapping of human chondroitin 4-sulfotransferase, whose expression pattern in human tissues is different from that of chondroitin 6-sulfotransferase. *J. Biochem.* **128**, 763-770

32. Kang, H. G., Evers, M. R., Xia, G., Baenziger, J. U., and Schachner, M. (2002) Molecular cloning and characterization of chondroitin-4-O-sulfotransferase-3. A novel member of the HNK-1 family of sulfotransferases. *J. Biol. Chem.* **277**, 34766-34772

33. Pacheco, B., Maccarana, M., and Malmstrom, A. (2009) Dermatan 4-O-sulfotransferase 1 is pivotal in the formation of iduronic acid blocks in dermatan sulfate. *Glycobiology.* **19**, 1197-1203

34. Ohtake, S., Ito, Y., Fukuta, M., and Habuchi, O. (2001) Human N-acetylgalactosamine 4-sulfate 6-O-sulfotransferase cDNA is related to human B cell recombination activating gene-associated gene. *J. Biol. Chem.* **276**, 43894-43900

35. Ohtake, S., Kimata, K., and Habuchi, O. (2003) A unique nonreducing terminal modification of chondroitin sulfate by N-acetylgalactosamine 4-sulfate 6-o-sulfotransferase. *J. Biol. Chem.* **278**, 38443-38452

36. Esko, J. D., and Selleck, S. B. (2002) Order out of chaos: assembly of ligand binding sites in heparan sulfate. *Annu. Rev. Biochem.* **71**, 435-471

37. Shukla, D., Liu, J., Blaiklock, P., Shworak, N. W., Bai, X., Esko, J. D., Cohen, G. H., Eisenberg, R. J., Rosenberg, R. D., and Spear, P. G. (1999) A novel role for 3-O-sulfated heparan sulfate in herpes simplex virus 1 entry. *Cell.* **99**, 13-22

38. Xia, G., Chen, J., Tiwari, V., Ju, W., Li, J. P., Malmstrom, A., Shukla, D., and Liu, J. (2002) Heparan sulfate 3-O-sulfotransferase isoform 5 generates both an antithrombin-binding site and an entry receptor for herpes simplex virus, type 1. *J. Biol. Chem.* **277**, 37912-37919

39. Xu, D., Tiwari, V., Xia, G., Clement, C., Shukla, D., and Liu, J. (2005) Characterization of heparan sulphate 3-O-sulphotransferase isoform 6 and its role in assisting the entry of herpes simplex virus type 1. *Biochem. J.* **385**, 451-459

40. Pikas, D. S., Eriksson, I., and Kjellen, L. (2000) Overexpression of different isoforms of glucosaminyl N-deacetylase/N-sulfotransferase results in distinct heparan sulfate N-sulfation patterns. *Biochemistry.* **39**, 4552-4558

41. van den Born, J., Pikas, D. S., Pisa, B. J., Eriksson, I., Kjellen, L., and Berden, J. H. (2003) Antibody-based assay for N-deacetylase activity of heparan sulfate/heparin N-deacetylase/N-sulfotransferase (NDST): novel characteristics of NDST-1 and -2. *Glycobiology.* **13**, 1-10

42. Baietti, M. F., Zhang, Z., Mortier, E., Melchior, A., Degeest, G., Geeraerts, A., Ivarsson, Y., Depoortere, F., Coomans, C., Vermeiren, E., Zimmermann, P., and David, G. (2012) Syndecan-syntenin-ALIX regulates the biogenesis of exosomes. *Nat. Cell. Biol.* **14**, 677-685

43. Duncan, M. B., Liu, M., Fox, C., and Liu, J. (2006) Characterization of the N-deacetylase domain from the heparan sulfate N-deacetylase/N-sulfotransferase 2. *Biochem. Biophys. Res. Commun.* **339**, 1232-1237

44. Aikawa, J., and Esko, J. D. (1999) Molecular cloning and expression of a third member of the heparan sulfate/heparin GlcNAc N-deacetylase/ N-sulfotransferase family. *J. Biol. Chem.* **274**, 2690-2695

45. Aikawa, J., Grobe, K., Tsujimoto, M., and Esko, J. D. (2001) Multiple isozymes of heparan sulfate/heparin GlcNAc N-deacetylase/GlcN N-sulfotransferase. Structure and activity of the fourth member, NDST4. *J. Biol. Chem.* **276**, 5876-5882

46. Tornberg, J., Sykiotis, G. P., Keefe, K., Plummer, L., Hoang, X., Hall, J. E., Quinton, R., Seminara, S. B., Hughes, V., Van Vliet, G., Van Uum, S., Crowley, W. F., Habuchi, H., Kimata, K., Pitteloud, N., and Bulow, H. E. (2011) Heparan sulfate 6-O-sulfotransferase 1, a gene involved in extracellular sugar modifications, is mutated in patients with idiopathic hypogonadotrophic hypogonadism. *Proc. Natl. Acad. Sci. U. S. A.* **108**, 11524-11529

47. Esko, J. D., and Selleck, S. B. (2002) Order out of chaos: assembly of ligand binding sites in heparan sulfate. *Annu Rev Biochem* **71**, 435-471

48. Habuchi, H., Miyake, G., Nogami, K., Kuroiwa, A., Matsuda, Y., Kusche-Gullberg, M., Habuchi, O., Tanaka, M., and Kimata, K. (2003) Biosynthesis of heparan sulphate with diverse structures and functions: two alternatively spliced forms of human heparan sulphate 6-O-sulphotransferase-2 having different expression patterns and properties. *Biochem. J.* **371**, 131-142

49. Paganini, L., Hadi, L. A., Chetta, M., Rovina, D., Fontana, L., Colapietro, P., Bonaparte, E., Pezzani, L., Marchisio, P., Tabano, S. M., Costanza, J., Sirchia, S. M., Riboni, L., Milani, D., and Miozzo, M. (2019) A HS6ST2 gene variant associated with X-linked intellectual disability and severe myopia in two male twins. *Clin. Genet.* **95**, 368-374

50. Sugahara, K., and Kitagawa, H. (2000) Recent advances in the study of the biosynthesis and functions of sulfated glycosaminoglycans. *Curr. Opin. Struct. Biol.* **10**, 518-527
